# SLMP53-2 Restores Wild-Type-Like Function to Mutant p53 through Hsp70: Promising Activity in Hepatocellular Carcinoma

**DOI:** 10.3390/cancers11081151

**Published:** 2019-08-10

**Authors:** Sara Gomes, Bartolomeo Bosco, Joana B. Loureiro, Helena Ramos, Liliana Raimundo, Joana Soares, Nair Nazareth, Valentina Barcherini, Lucília Domingues, Carla Oliveira, Alessandra Bisio, Silvano Piazza, Matthias R. Bauer, João P. Brás, Maria Inês Almeida, Célia Gomes, Flávio Reis, Alan R. Fersht, Alberto Inga, Maria M. M. Santos, Lucília Saraiva

**Affiliations:** 1 LAQV/REQUIMTE, Department of Biological Sciences, Laboratory of Microbiology, Faculty of Pharmacy, University of Porto, Rua de Jorge Viterbo Ferreira 228, 4050-313 Porto, Portugal; 2 Department of Cellular, Computational and Integrative Biology (CIBIO), University of Trento, Via Sommarive 9, 38123 Trento, Italy; 3 Research Institute for Medicines (iMed.ULisboa), Faculty of Pharmacy, University of Lisbon, Av. Prof. Gama Pinto, 1649-003 Lisboa, Portugal; 4 CEB—Centre of Biological Engineering, University of Minho, Campus de Gualtar, 4710-057 Braga, Portugal; 5 Medical Research Council, Laboratory of Molecular Biology, Francis Crick Avenue, Cambridge CB2 0QH, UK; 6 i3S—Institute for Research and Innovation in Health, University of Porto, Rua Alfredo Allen 208, 4200-135 Porto, Portugal; 7 INEB—Institute of Biomedical Engineering, University of Porto, Rua Alfredo Allen 208, 4200-135 Porto, Portugal; 8 Institute of Pharmacology & Experimental Therapeutics, Coimbra Institute for Clinical and Biomedical Research (iCBR), Faculty of Medicine, CNC.IBILI Consortium & CIBB Consortium, University of Coimbra, 3000-548 Coimbra, Portugal

**Keywords:** anticancer therapeutics, hepatocellular carcinoma, Hsp70, mutant p53, tryptophanol-derived oxazoloisoindolinone

## Abstract

Half of human cancers harbor *TP53* mutations that render p53 inactive as a tumor suppressor. In these cancers, reactivation of mutant p53 (mutp53) through restoration of wild-type-like function constitutes a valuable anticancer therapeutic strategy. In order to search for mutp53 reactivators, a small library of tryptophanol-derived oxazoloisoindolinones was synthesized and the potential of these compounds as mutp53 reactivators and anticancer agents was investigated in human tumor cells and xenograft mouse models. By analysis of their anti-proliferative effect on a panel of p53-null NCI-H1299 tumor cells ectopically expressing highly prevalent mutp53, the compound SLMP53-2 was selected based on its potential reactivation of multiple structural mutp53. In mutp53-Y220C-expressing hepatocellular carcinoma (HCC) cells, SLMP53-2-induced growth inhibition was mediated by cell cycle arrest, apoptosis, and endoplasmic reticulum stress response. In these cells, SLMP53-2 restored wild-type-like conformation and DNA-binding ability of mutp53-Y220C by enhancing its interaction with the heat shock protein 70 (Hsp70), leading to the reestablishment of p53 transcriptional activity. Additionally, SLMP53-2 displayed synergistic effect with sorafenib, the only approved therapy for advanced HCC. Notably, it exhibited potent antitumor activity in human HCC xenograft mouse models with a favorable toxicological profile. Collectively, SLMP53-2 is a new mutp53-targeting agent with promising antitumor activity, particularly against HCC.

## 1. Introduction

As the most frequently inactivated tumor suppressor in human tumors, p53 has attracted great interest as an anticancer therapeutic target. p53 is a transcription factor that regulates several cellular processes, including apoptosis, cell cycle, and DNA-damage repair [1]. Over half of human tumors harbor *TP53* mutations, leading not only to the impairment of p53 tumor-suppressive activity, but also to gain-of-function that promotes tumor growth, dissemination, and chemoresistance [2]. Most p53 mutations occur within the DNA-binding domain (DBD). The amino acid substitutions in mutant p53 (mutp53) may lead to substantial protein unfolding (structural mutations; e.g., R175H, Y220C, G245S), or to loss of DNA contacts, with no significant structural destabilization (contact mutations; e.g., R280K, R273H) [2,3]. Regardless of the type, mutp53 loses transcriptional, and therefore tumor-suppressive, activity. Hence, restoring wild-type (wt)-like activity to mutp53 is an appealing anticancer therapeutic strategy. In fact, extensive efforts have been made in the search for mutp53 reactivators [4]. Among the mutp53 reactivators disclosed to date, distinct molecular mechanisms have been reported. While some reactivators bind to mutp53, others display indirect reactivation mechanisms by targeting proteins like heat shock protein 40 (Hsp40), which promotes mutp53 refolding and subsequent re-establishment of DNA-binding ability [5]. Although several small molecule mutp53 reactivators have been reported to date, only APR-246 (PRIMA-1^MET^) and COTI-2 are currently undergoing clinical trials [5].

Primary liver cancer presented the sixth highest incidence worldwide in 2018 [6], which is predicted to increase in the upcoming years [7]. Hepatocellular carcinoma (HCC), the most common histologic type of primary liver cancer, is associated with unfavorable prognosis, mainly due to high chemoresistance and recurrence rates [8,9]. The majority of patients are diagnosed at advanced-or terminal-stage, and available therapeutic options are restricted to symptomatic treatment or sorafenib, a multi-target kinase inhibitor [8,9]. Nonetheless, the increase of median overall survival of HCC patients treated with sorafenib is only 2.8 months [8], making the identification of effective therapeutic alternatives a high priority. About 30% of HCC harbor p53 mutations, correlating with increased invasiveness, recurrence, and lower survival rates [10,11]. This makes mutp53 a privileged therapeutic target in HCC. Herein, we unveil the new mutp53 reactivator SLMP53-1 with great potential as an anticancer agent, particularly against HCC.

## 2. Results

### 2.1. SLMP53-2 Displays mutp53-Dependent Growth Inhibitory Effect in Human Tumor Cells, Leading to Cell Cycle Arrest, Apoptosis and Endoplasmic Reticulum (ER) Stress

**Figure 1:**
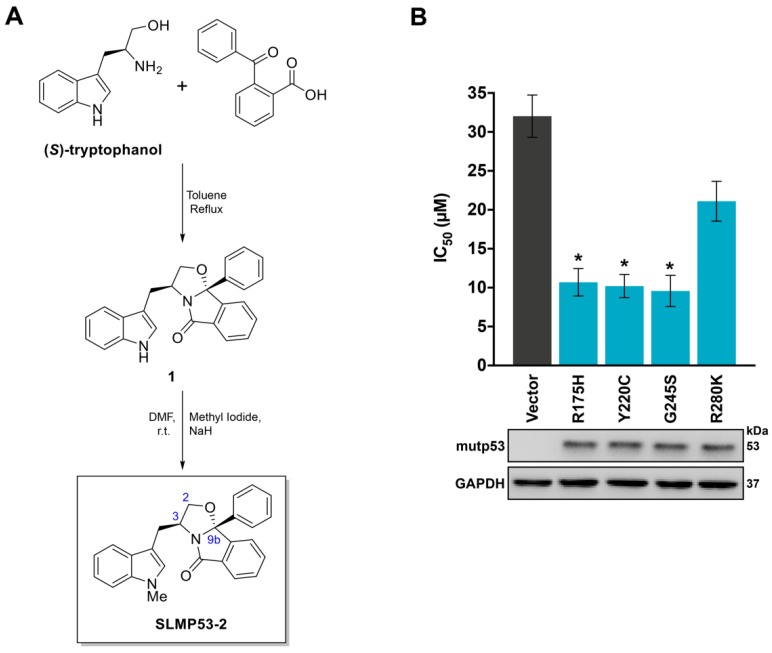
Growth inhibitory effect of SLMP53-2 in human tumor cells is dependent on structural mutp53. (**A**) Chemical synthesis of SLMP53-2. (**B**) IC_50_ values of SLMP53-2, in p53-null H1299 cells transfected with pcDNA3 expressing different mutp53 or empty vector, were determined by SRB assay after 48h treatment with 3.12–50 µM SLMP53-2; data are mean ± SEM (*n* = 5); values significantly different from pcDNA3-Empty: * *p* < 0.05, one-way ANOVA with Dunnett’s multiple comparison test. Mutp53 expression was confirmed by western blot; GAPDH was used as a loading control. Immunoblots represent one of three independent experiments.

In our previous work, the tryptophanol-derived oxazoloisoindolinone SLMP53-1 was identified as a reactivator of mutp53-R280K with in vivo p53-dependent anti-tumor activity [12]. In order to search for new mutp53 reactivators, a small library of SLMP53-1 derivatives was synthesized. The activity of the compounds as potential mutp53 reactivators was investigated by analysis of their anti-proliferative effect on p53-null NCI-H1299 tumor cells ectopically expressing four prevalent mutp53 forms. By sulforhodamine B (SRB) assay, the compound SLMP53-2 (Figure 1A) was selected based on its marked reduction of the IC_50_ values in NCI-H1299 cells expressing mutp53-R175H, -Y220C, or -G245S, compared to cells transfected with the empty vector (Figure 1B). These results evidenced the ability of SLMP53-2 to reactivate multiple structural mutp53. Interestingly, they also showed a distinct selectivity of SLMP53-1 and -2 towards different mutp53. In fact, unlike SLMP53-1 [12], SLMP53-2 was able to activate mutp53-Y220C, not significantly interfering with mutp53-R280K activity (Figure 1B).

**Figure 2:**
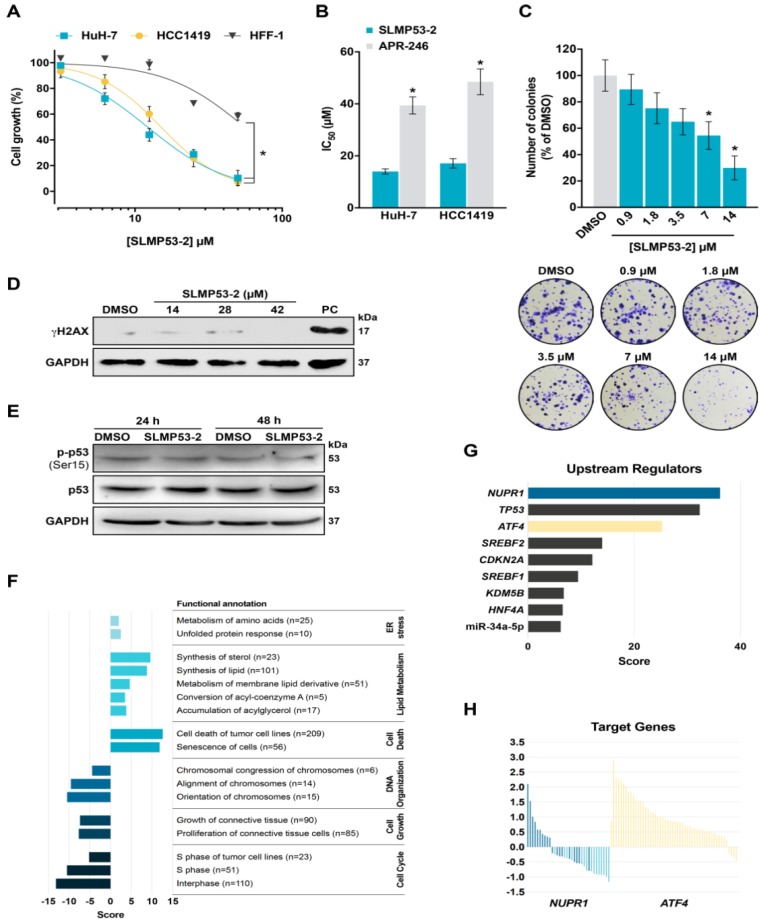
SLMP53-2 inhibits the growth of mutp53-Y220C-expressing tumor cells with no genotoxicity, leading to the differential expression of genes involved in cell cycle and death, lipid metabolism, and endoplasmic reticulum (ER) stress. (**A**) Concentration-response curves for SLMP53-2 in human non-tumoral HFF-1 and tumor mutp53-Y220C-expressing HuH-7 and HCC1419 cells, analyzed by sulforhodamine B (SRB) assay after 48 h treatment with 3.12–50 µM SLMP53-2. Data are mean ± SEM (*n* = 5); * *p* < 0.05, extra sum-of-squares F test. (**B**) IC_50_ values of SLMP53-2 and APR-246 in HuH-7 and HCC1419 cells were determined by SRB assay after 48 h treatment with 3.12–50 µM SLMP53-2 or APR-246. Data are mean ± SEM (*n* = 5); * *p* < 0.05, two-way ANOVA with Sidak’s multiple comparison test. (**C**) Effect of SLMP53-2 in HuH-7 cell colony formation, analyzed after 14 days incubation with SLMP53-2; a representative experiment is shown. Data are mean ± SEM (*n* = 5); values significantly different from DMSO: * *p* < 0.05, one-way ANOVA with Dunnett’s multiple comparison test. (**D**) Levels of γH2AX in HuH-7 cells treated with SLMP53-2; 50 µM etoposide was used as a positive control (PC). (**E**) Levels of mutp53 phosphorylation at Ser15 in HuH-7 cells treated with 14 µM SLMP53-2. In (**D**,**E**), immunoblots represent one of three independent experiments; glyceraldehyde 3-phosphate dehydrogenase (GAPDH) was used as a loading control. (**F**) Top enriched biological pathways grouped by broad categories based on Ingenuity Pathway Analysis (IPA) starting from the dataset of differentially expressed genes (DEGs) from HuH-7 cells treated with 28 µM SLMP53-2. The number of features for each functional annotation is given in parenthesis. The score combines the log10 *p*-value and predicted pathway activation or repression status of the corresponding pathway/process, respectively for positive and negative score. The different colors correspond to the different functional annotation categories. (**G**) Top scoring upstream regulators inferred from the same gene expression dataset. The score is the log10 *p*-value of the predicted activation status. (**H**) Gene expression changes of *NUPR1* (blue) and *ATF4* (yellow) target genes in SLMP53-2-treated HuH-7 cells.

For an in-depth analysis of the molecular mechanism of SLMP53-2, we focused on mutp53-Y220C, which is known to have a druggable hydrophobic pocket [13]. First, the growth inhibitory effect of SLMP53-2 against HuH-7 and HCC1419 cells endogenously expressing mutp53-Y220C was evaluated by SRB assay. As expected, SLMP53-2 inhibited the growth of both tumor cells with similar IC_50_ values (Figure 2A), and higher potency than APR-246 (Figure 2B). Notably, the growth inhibitory activity of SLMP53-2 against non-tumoral HFF-1 cells (IC_50_ of 50 µM) was significantly lower compared to tumor cells (Figure 2A).

In HuH-7 cells, SLMP53-2 displayed a concentration-dependent growth inhibitory effect on colony formation (Figure 2C). We next assessed whether the growth inhibitory effect of SLMP53-2 in HuH-7 cells was associated with DNA damage. However, unlike the positive control (50 µM etoposide), no induction of H2AX phosphorylation (γH2AX) was detected after 14, 28, or 42 µM SLMP53-2 treatments (Figure 2D). Consistently, 14 µM SLMP53-2 did not promote mutp53 phosphorylation at Ser15 (Figure 2E), which is a major p53 phosphorylation site in response to genotoxic stresses [14].

A microarray analysis (GSE124021) indicated that 28 and 42 µM SLMP53-2 led to the differential expression of more than 700 genes (four replicates for each treatment and DMSO (dimethyl sulfoxide) control, adjusted *p* value < 0.05, log2 fold change >0.6 and <−0.6) in HuH-7 cells (Table S1, Figures S1 and S2). Pathway and upstream regulator analyses (Ingenuity Pathway, Metascape and Enrichr) identified signatures consistent with downregulation of cell cycle progression, upregulation of lipid metabolism and cell death, and ER stress induction (Figure 2F–H, Figures S1 and S2 and Table S2). In SLMP53-2-treated HuH-7 cells, *NUPR1*, *TP53* and *ATF4* were among the top scoring upstream regulators inferred from the gene expression dataset. *NUPR1* is an *ATF4* target and can contribute to ER stress responses, cell proliferation and apoptosis [15]. Gene expression changes showed that most *ATF4* target genes were upregulated, while the majority of *NUPR1* targets were downregulated (Figure 2H). This was expected as several *NUPR1* targets are involved in cell cycle and proliferation. Interestingly, downregulated genes were enriched for targets of miR-34a (Figure S2), which is a well-established p53-inducible microRNA [16]. Gene expression changes identified by microarray analysis were confirmed by qPCR for selected genes (Figure S2). The gene expression signature had similarities with molecules inducing apoptosis, autophagy or inhibiting the proteasome, based on Connectivity Map (Figure S3 and Table S3).

**Figure 3:**
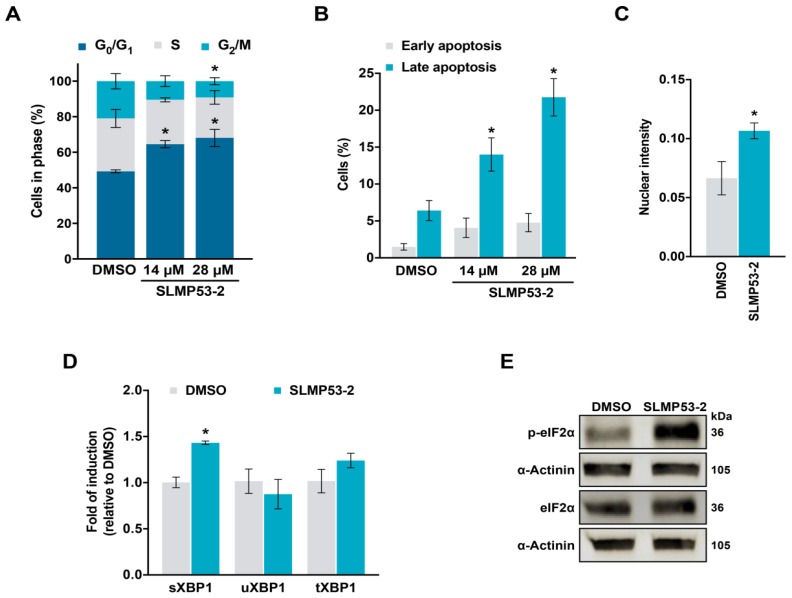
Growth inhibitory effect of SLMP53-2 in hepatocellular carcinoma (HCC) cells is associated with cell cycle arrest, apoptosis, and ER stress. (**A**) Effect of SLMP53-2 on cell cycle progression of HuH-7 cells after 48 h treatment; values significantly different from DMSO: * *p* < 0.05, two-way ANOVA with Dunnett’s multiple comparison test. (**B**) Effect of SLMP53-2 on apoptosis of HuH-7 cells, after 72 h treatment. Data are mean ± SEM (*n* = 5); values significantly different from DMSO: * *p* < 0.05, one-way ANOVA with Dunnett’s multiple comparison test. (**C**) Quantification of nuclear fluorescence intensity of XBP1 in HuH-7 cells after 24 h treatment with 28 µM SLMP53-2. Data are mean ± SEM (*n* = 3); values significantly different from DMSO: * *p* < 0.05, unpaired Student’s *t*-test. (**D**) mRNA levels of spliced (sXBP1), unspliced (uXBP1), and total XBP1 (tXBP1) in HuH-7 cells after 24 h treatment with 28 µM SLMP53-2, determined by RT-qPCR; fold of induction is relative to DMSO. Data are mean ± SEM (*n* = 3); values significantly different from DMSO: * *p* < 0.05, two-way ANOVA with Dunnett’s multiple comparison test. (**E**) Protein levels of phosphorylated and total eIF2α in HuH-7 cells, after 24 h treatment with 28 µM SLMP53-2. Immunoblots represent one of three independent experiments; α-actinin was used as a loading control.

In accordance with microarray data, 14 and 28 µM SLMP53-2 induced G0/G1-phase cell cycle arrest (Figure 3A) and apoptosis (Figure 3B), in HuH-7 cells. Moreover, consistently with the induction of an ER stress response, 28 µM SLMP53-2 increased the levels of XBP1 nuclear protein (Figure 3C and Figure S4), spliced XBP1 (sXBP1) mRNA (Figure 3D), and phosphorylated eIF2α (Figure 3E).

### 2.2. SLMP53-2 Restores WT-Like Conformation and Transcriptional Activity to mutp53-Y220C in HCC Cells

**Figure 4:**
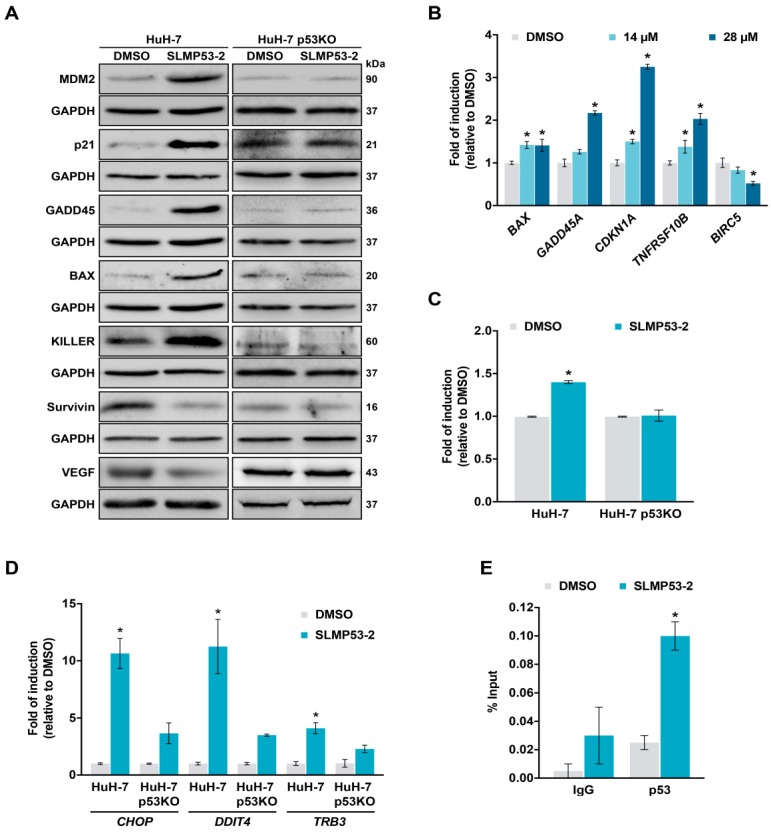
SLMP53-2 induces p53 transcriptional targets, including miR-34a and genes involved in ER stress response, and restores DNA-binding ability to mutp53-Y220C in HCC cells. (**A**) Protein levels of p53 target genes in parental and p53KO HuH-7 cells, after 16 h (p21), 24 h (survivin, VEGF) or 48 h (MDM2, GADD45, BAX, KILLER) treatment with 14 µM SLMP53-2. Immunoblots represent one of three independent experiments; GAPDH was used as a loading control. (**B**) mRNA levels of p53 target genes, in HuH-7 cells, after 24 h treatment with 14 and 28 µM SLMP53-2, determined by RT-qPCR; fold of induction is relative to DMSO. (**C**) miR-34a levels in parental and p53KO HuH-7 cells, after 24 h treatment with 28 µM SLMP53-2, determined by RT-qPCR; fold of induction is relative to DMSO. (**D**) mRNA levels of genes involved in the ER stress response, in parental and p53KO HuH-7 cells, after 24 h treatment with 28 µM SLMP53-2, determined by RT-qPCR; fold of induction is relative to DMSO. (**E**) Analysis of the occupancy of mutp53-Y220C on the p21 promoter in HuH-7 cells, determined by ChIP, after 24 h treatment with 28 µM SLMP53-2 or DMSO; immunoprecipitation was performed with an anti-p53 antibody (p53) or an anti-mouse IgG as a negative control (IgG); the enrichment of DNA fragments was analyzed by RT-qPCR using site-specific primers. In **A**–**E**, data are mean ± SEM (*n* = 3); values significantly different from DMSO (**B**–**E**): * *p* < 0.05, two-way ANOVA with Dunnett’s (**B**,**E**) or Sidak’s (**D**) multiple comparison test; * *p* < 0.05, unpaired Student’s *t*-test (**C**).

We next evaluated the effect of SLMP53-2 on the expression levels of several p53 target genes. By western blot, it was observed that 14 µM SLMP53-2 increased the protein levels of MDM2, p21, GADD45, BAX, and KILLER, while downregulating survivin and VEGF, in HuH-7 cells, an effect abolished in HuH-7 p53KO cells (Figure 4A). It is of note that in mutp53-Y220C-expressing HCC1419 cells, 14 µM SLMP53-2 also interfered with the protein levels of several p53 target genes, increasing MDM2, p21, and KILLER, and decreasing survivin and VEGF (Figure S5).

In HuH-7 cells, the regulation of p53 target genes by 14 and 28 µM SLMP53-2 was further confirmed at mRNA level with the upregulation of *BAX*, *GADD45*, *CDKN1A* (p21), and *TNFRSF10B* (KILLER), and the downregulation of *BIRC5* (survivin) (Figure 4B). Consistently with the microarray analysis, 28 µM SLMP53-2 also upregulated miR-34a in HuH-7, but not in HuH-7-p53KO cells (Figure 4C). Moreover, 28 µM SLMP53-2 significantly enhanced the expression of p53 target genes involved in the ER stress response, namely *CHOP* and *DDIT4*, as well as of *TRB3* (a *CHOP* target gene [17,18,19]) in HuH-7, but not in HuH-7-p53KO cells (Figure 4D). The reestablishment of mutp53-Y220C transcriptional activity was further supported by chromatin immunoprecipitation (ChIP). In fact, 28 µM SLMP53-2 significantly increased mutp53-Y220C occupancy at the p21 promoter (Figure 4E), reflecting the restoration of p53 DNA-binding ability.

**Figure 5:**
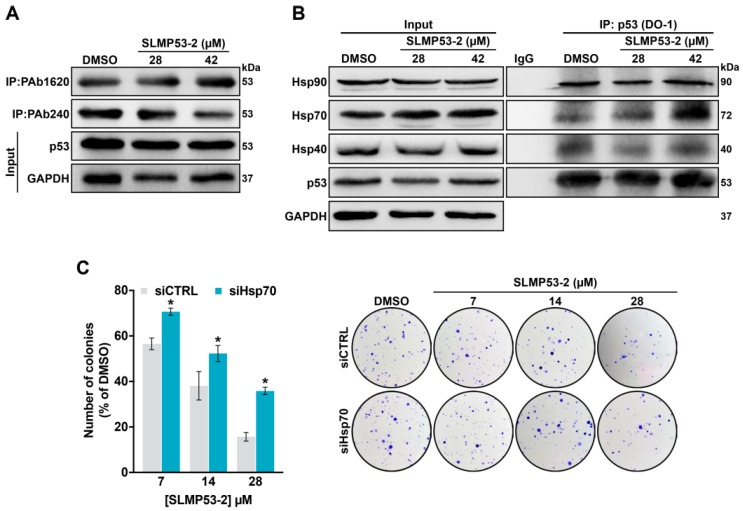
SLMP53-2 restores wt-like folding and promotes mutp53-Y220C interaction with Hsp70. (**A**) Immunoprecipitation of p53 in HuH-7 cells treated with 28 and 42 µM SLMP53-2 for 36 h, using the conformation-specific antibodies PAb240 (mut/unfolded) and PAb1620 (wt/folded), followed by immunoblotting with anti-p53 (DO-1) or anti-Hsp70 antibodies; whole cell lysate (input). Of note that labelling with PAb1620 in DMSO-treated cells may be explained by mutp53-Y220C temperature-sensitivity [20]. (**B**) Co-immunoprecipitation of Hsp40, Hsp70, and Hsp90 with p53 in HuH-7 cells treated with 28 and 42 µM SLMP53-2 or DMSO for 36 h, using anti-p53 antibody (DO-1), followed by immunoblotting with anti-Hsp40, anti-Hsp70, anti-Hsp90, and anti-p53 antibodies; whole cell lysate (input). (**C**) Effect of SLMP53-2 in the cell colony formation of HuH-7 cells transfected with siRNA against Hsp70 (siHsp70) or control siRNA (siCTRL), analysed after 14 days incubation with SLMP53-2; a representative experiment is shown. Data are mean ± SEM (*n* = 5); values significantly different from siCTRL: * *p* < 0.05, one-way ANOVA with Dunnett’s multiple comparison test. In (**A**,**B**), immunoblots represent one of three independent experiments; GAPDH was used as a loading control.

As a structural mutp53, we investigated the ability of SLMP53-2 to restore wt-like folding to mutp53-Y220C, through immunoprecipitation (IP) using PAb1620 (wt/folded) and PAb240 (mut/unfolded) conformation-specific antibodies. The results showed that 42 µM SLMP53-2 visibly increased the amount of p53 precipitated with PAb1620, while decreasing the amount of p53 precipitated with PAb240 (Figure 5A). Consistently, 42 µM SLMP53-2 visibly increased PAb1620 and decreased PAb240 immunofluorescence staining in HuH-7 cells (Figure S6).

### 2.3. Hsp70 is a Potential Mediator of mutp53-Y220C Reactivation by SLMP53-2

To determine the mechanism by which SLMP53-2 would affect mutp53-Y220C function, we checked whether SLMP53-2 could bind to this mutp53. However, despite the presence of a druggable hydrophobic pocket in mutp53-Y220C, no binding of SLMP53-2 to recombinant mutp53-Y220C DBD was detected by heteronuclear single-quantum coherence (HSQC)-NMR (Figure S7).

Chaperones from the Hsp40, Hsp70, and Hsp90 families have long been recognized as mutp53-binding partners capable of folding mutp53 to the wt-like conformation [21,22,23]. Therefore, we interrogated whether SLMP53-2 could reactivate mutp53-Y220C by promoting its interaction with these chaperones. To investigate potential interactions between mutp53-Y220C and Hsp40/Hsp70/ Hsp90, co-immunoprecipitation (co-IP) experiments were performed in HuH-7 cells. Conversely to that observed with Hsp40 and Hsp90, 42 µM SLMP53-2 visibly increased the amount of Hsp70 precipitated with p53 (Figure 5B), indicating an enhancement of Hsp70 binding to p53. Moreover, in Hsp70 siRNA silenced HuH-7 cells (Figure S8), the inhibitory effect of SLMP53-2 on colony formation was significantly reduced (Figure 5C). Therefore, SLMP53-2 may restore wt-like conformation to mutp53-Y220C through enhancement of its interaction with Hsp70.

### 2.4. SLMP53-2 Sensitizes HCC Cells to Sorafenib

**Figure 6:**
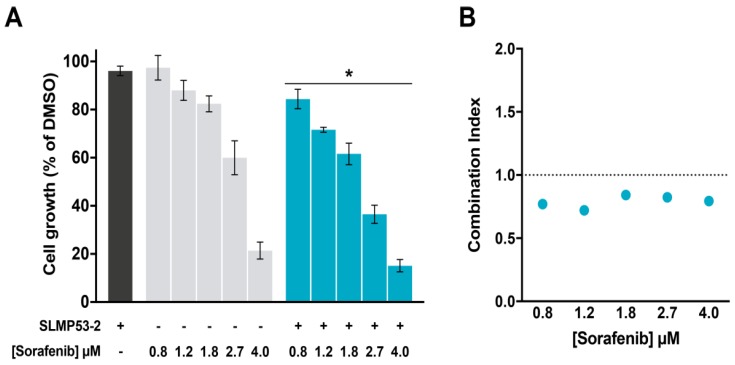
SLMP53-2 synergizes with sorafenib in HCC cells. HuH-7 cells were treated with increasing concentrations of sorafenib, alone and in combination with 1.5 µM SLMP53-2. (**A**) Cell growth was measured by SRB assay after 48 h treatment; growth obtained with control (DMSO) was set as 100%. Data are mean ± SEM (*n* = 4); values significantly different from chemotherapeutic alone: * *p* < 0.05, two-way ANOVA. (**B**) Combination index (CI) values calculated using the CompuSyn software for each combined treatment. CI < 1, synergy; 1 < CI < 1.1, additive effect; CI > 1.1, antagonism. CI values were calculated using a mean value effect (*n* = 4).

A potential synergistic association between SLMP53-2 and sorafenib was assessed by SRB assay, in HuH-7 cells. When combined with 1.5 µM SLMP53-2, sorafenib displayed significantly higher growth inhibitory effect compared to its effect as a single agent (Figure 6A). Consistently, a synergistic effect (CI < 1) was obtained for all tested sorafenib concentrations in combination with 1.5 µM SLMP53-2 (Figure 6B).

### 2.5. SLMP53-2 Displays In Vivo Anti-Tumor Activity in HCC Xenograft Mouse Models, with No Apparent Toxic Side Effects

The toxicity profile of SLMP53-2 was evaluated in Wistar rats subjected to five intraperitoneal administrations of 50 mg/kg SLMP53-2 or vehicle, followed by analysis of hematological and biochemical parameters from blood samples (Table S4). Although a slight reduction of the red cell distribution width and mean platelet volume was observed upon SLMP53-2 treatment, these values were within the reference range [24]. Therefore, no undesirable hematological and biochemical toxicity was induced by SLMP53-2 in vivo.

**Figure 7:**
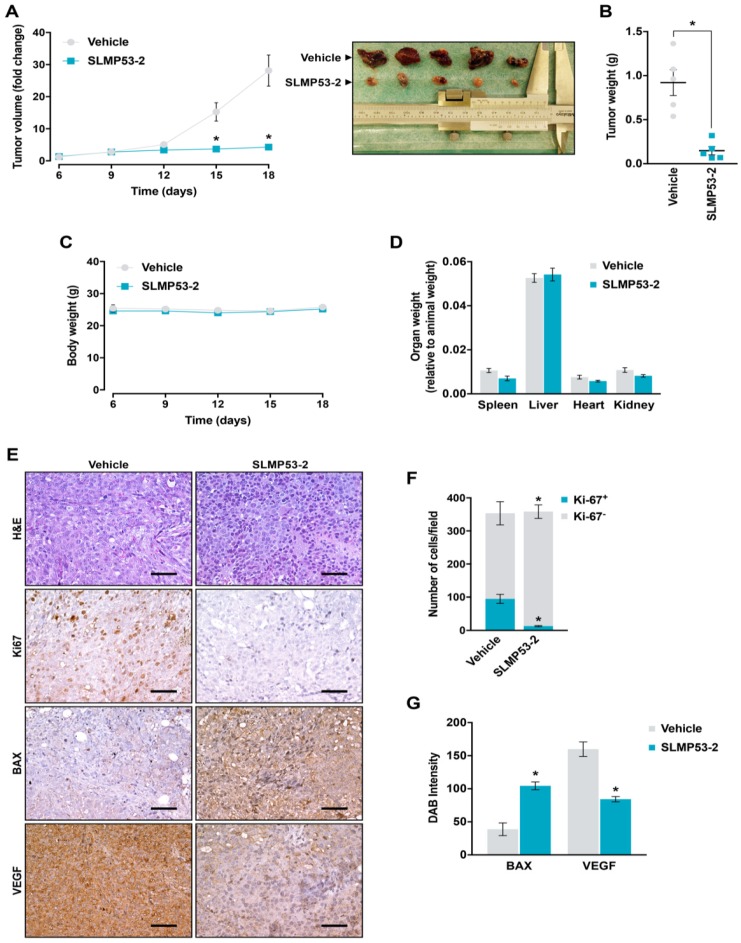
In vivo anti-tumor activity of SLMP53-2. Swiss nude mice carrying HuH-7 xenografts were treated with 50 mg/kg SLMP53-2 or vehicle, by intraperitoneal injection twice a week, for a total of five administrations. (**A**) Tumor volume curves of mice carrying HuH-7 xenografts treated with SLMP53-2 or vehicle; image of vehicle and SLMP53-2-treated tumors at the end of treatment. (**B**) Tumor weights measured at the end of the experiment; value significantly different from vehicle: * *p* < 0.05, unpaired Student’s *t*-test. (**C**) Mice body weight during treatment with SLMP53-2 or vehicle. (**D**) Weight of spleen, liver, heart and kidneys, relative to animal weight, in animals treated with SLMP53-2 or vehicle. (**E**) Representative images of Ki-67, BAX, and VEGF detection in tumor tissues of HuH-7 xenografts treated with SLMP53-2 or vehicle, collected at the end of treatment (scale bar = 20 μm; magnification = 200×); hematoxylin and eosin (H&E). (**F**,**G**) Quantification of immunohistochemistry of HuH-7 xenograft tumor tissues treated with SLMP53-2 or vehicle; in (**F**), quantification of the number of Ki-67 positive and negative cells (*n* = 5); in (**G**), BAX and VEGF staining were quantified by evaluation of 3,3′-diaminobenzidine (DAB) intensity (*n* = 5); values significantly different from vehicle: * *p* < 0.05, unpaired Student’s *t-*test. In (**A**,**D**), data are mean ± SEM (*n* = 5). In (**A**) and (**F**), values significantly different from vehicle: * *p* < 0.05, two-way ANOVA with Sidak’s multiple comparison test. In (**C**,**D**), values not significantly different from vehicle: *p* > 0.05, two-way ANOVA with Sidak’s multiple comparison test.

To evaluate the in vivo anti-tumor activity of SLMP53-2, five intraperitoneal administrations of 50 mg/kg SLMP53-2 or vehicle were performed in nude mice carrying HuH-7 xenografts. A pronounced reduction of tumor volume (Figure 7A) and weight (Figure 7B) was observed in SLMP53-2-treated mice compared to vehicle. Moreover, nude mice showed no significant variation of body weight throughout the experiment (Figure 7C), and no significant differences were observed between the weight of spleen, liver, heart, and kidneys of SLMP53-2-treated mice and vehicle (Figure 7D). These results further supported a favorable therapeutic index of SLMP53-2 in vivo. Additionally, the immunohistochemistry (IHC) staining of tumor sections revealed that SLMP53-2 decreased Ki-67 and VEGF, and increased BAX staining (Figure 7E–G), compared to vehicle. Altogether, these results unveiled a potent in vivo anti-tumor activity of SLMP53-2 through induction of apoptosis and inhibition of cell proliferation and angiogenesis.

## 3. Discussion

The advances in understanding cancer pathobiology have supported the crucial role of mutp53 in all hallmarks of cancer, rendering mutp53 reactivation one of the most promising therapeutic strategies in cancer treatment. The reactivation of mutp53 also poses a great opportunity in combination therapy with conventional chemotherapies known to trigger cancer cell death through an active p53-dependent pathway. Nonetheless, drugging mutp53 has shown to be a challenging task, with no mutp53 reactivators still available for clinical use. In fact, although the potential of reactivating wt-like function to mutp53 with canonical genes transcription has been proven, it does not always translate into halting tumor growth [1,2,4,5].

In this work, we report the identification of SLMP53-2 as a novel reactivator of mutp53, with p53-dependent growth inhibitory effect on human tumor cells through induction of cell cycle arrest and apoptosis. Interestingly, SLMP53-2 stimulates the ER stress response by inducing the expression of several ER stress markers, including the p53-target gene *CHOP*, which triggers ER stress-related cell death [25]. ER stress occurs when the tightly-regulated protein folding environment of the ER is disrupted, causing accumulation of unfolded proteins. This triggers the unfolded protein response (UPR), a mechanism intended to re-establish ER homeostasis. When the UPR fails to restore ER homeostasis, cell death is triggered [25]. In fact, ER stress exacerbation for cell death induction has been proposed as a relevant anticancer therapeutic strategy, specifically against HCC [26]. Particularly, the prodrug of thapsigargin, mipsagargin (a known ER stress inducer through interference with ER calcium levels) was tested in patients with solid tumors, including HCC [27]. Moreover, zebularine (a DNA methyltransferase inhibitor) has been reported to induce p53-dependent ER stress-mediated cell death in colorectal cancer cells [28]. Interestingly, the mutp53 reactivators APR-246 and PK11007 have also been reported to induce ER stress-mediated cell death in mutp53-expressing tumor cells [29,30].

Herein, SLMP53-2 is also shown to restore wt-like folding and DNA-binding ability to mutp53-Y220C. In fact, SLMP53-2 reactivates p53 transcriptional activity, regulating the expression of several p53 transcriptional targets involved in cell cycle arrest (p21, GADD45), apoptosis (BAX, KILLER, survivin), ER stress response (CHOP, DDIT4), and angiogenesis (VEGF). Interestingly, SLMP53-2 causes p53-dependent reduction of the expression levels of survivin, which is frequently overexpressed in HCC, correlating with increased invasion and metastasis, and decreased overall and relapse-free survival [31,32,33]. Additionally, SLMP53-2 upregulates miR-34a, a p53 transcriptional target. Consistently, the microarray data revealed that SLMP53-2 leads to downregulation of several miR-34a targets. miR-34a is a crucial tumor suppressor that controls cellular processes including proliferation, apoptosis, senescence, and stemness [34]. This is of particular interest in HCC, in which miR-34a downregulation is common and frequently associated with invasion and metastasis [35]. Consistently, MRX34, a liposomal miR-34a mimic, has displayed promising anti-tumor activity in orthotopic HCC models [36], having recently completed phase I clinical trials for advanced solid tumors, including HCC [37].

Despite its inability to bind to mutp53-Y220C, SLMP53-2 enhances the mutp53-Y220C interaction with chaperone Hsp70. In fact, Hsp70 is a known mutp53 binding partner, leading to its refolding and protein stabilization, with subsequent restoration of DNA-binding and transcriptional activity [21,22,23]. A similar mutp53 reactivation mechanism was recently reported for chetomin, which restores wt-like function to mutp53-R175H by enhancing its interaction with Hsp40 [38]. Interestingly, the requirement of Hsp70 for mutp53-R175H stabilization, and its contribution to the pancreatic cells malignant transformation have been recently reported [39]. However, these observations were conducted in non-tumoral cells. A cell context-dependence of the Hsp70 effect on mutp53 might justify the distinct observations made in the current work.

The anti-proliferative effect of SLMP53-2 on p53-null tumor cells ectopically expressing four prevalent mutp53 forms supports its ability to reactivate structural mutp53 forms, including R175H, Y220C, and G245S. These results are reinforced by the capability of SLMP53-2 to restore mutp53-Y220C transcriptional activity in HCC cells endogenously expressing this mutp53. The activation of mutp53-Y220C transcriptional activity by SLMP53-2 was also evidenced in breast tumor cells expressing this mutp53, indicating its potential to reactivate mutp53 in distinct tumor cell types. It is also worth noting that SLMP53-2 has antitumor activity against wtp53-expressing tumor cells, namely HCC HepG2 cells (IC_50_ of 12.5 ± 0.8 µM; Figure S10A). In fact, a wtp53-dependent growth inhibitory effect could be evidenced for SLMP53-2, which showed a two-fold reduction in the IC_50_ value in HCT116 colon tumor cells expressing wtp53 (IC_50_ of 8.4 ± 1.1 µM) compared to its isogenic p53-null derivative (IC_50_ of 17.7 ± 2.3 µM) (Figure S10B). This is consistent with the proposed mechanism of action of SLMP53-2, since the stabilization of wtp53 by Hsp70 has also been reported [22].

In our previous work, SLMP53-1 was identified as a reactivator of mutp53 [12]. Despite being chemical derivatives, several structural differences can be found between SLMP53-1 and -2. In particular, SLMP53-2 has a phenyl instead of a methyl group in position 9b, and its indole nitrogen is protected with a methyl group. It is well-known that small structural modifications can completely change the activity of a compound on a specific target, since with the introduction of a certain group, the compound may no longer fit into the pocket of the target protein losing its activity. Moreover, it is currently accepted that structurally related drugs often act against distinct targets. In fact, only a small number of drugs that share the same or overlapping targets are structurally related [39]. As such, although both SLMP53-1 and -2 act as mutp53 reactivators, the inability of SLMP53-1 to reactivate the structural mutp53-Y220C, having effect on contact mutp53-R280K, allow us to predict a distinct mechanism of mutp53 reactivation. This hypothesis is supported by the inability of SLMP53-1 to enhance the Hsp70 interaction with mutp53-Y220C in HuH-7 cells (Figure S11), and it may explain their capability to reactivate distinct mutp53, as well as their distinct growth inhibitory activity towards tumor cells. Considering the relevance of mutp53 in the HCC pathogenesis and the lack of therapeutic alternatives for this cancer [10,11], this work also paves the way to a potential application of SLMP53-2 (alone or in combination therapy) in HCC treatment. Moreover, besides its growth inhibitory activity against HCC cells (superior to that of APR-246, currently in phase Ib/II clinical trials [5]), SLMP53-2 also sensitizes HCC cells to sorafenib, the only drug currently approved for the treatment of advanced HCC [8,9]. Importantly, in the HCC xenograft mouse model, SLMP53-2 displays pronounced antitumor activity associated with induction of apoptosis and inhibition of cell proliferation and angiogenesis. Interestingly, the crucial role of angiogenesis in HCC development and dissemination has been widely explored for the development of targeted therapies [40]. In fact, antiangiogenic agents, like sorafenib, have been the only targeted drugs with positive results in clinical trials for HCC [40]. Notably, SLMP53-2 also displays a favorable in vivo toxicological profile, with no significant alteration of toxicological parameters related to liver function. This is of particular importance in HCC, as patients often display liver cirrhosis that can lead to impaired liver function and consequently increased drug toxicity [41].

## 4. Materials and Methods

### 4.1. Compounds and Antibodies

Sorafenib, etoposide, and APR-246 were purchased from Santa Cruz Biotechnology (Heidelberg, Germany), Calbiochem (VWR, Carnaxide, Portugal), and Sigma-Aldrich (Sintra, Portugal), respectively. All antibodies are listed in Table S5.

### 4.2. Chemical Synthesis of SLMP53-2

#### 4.2.1. General Methods

All reagents and solvents were obtained from commercial suppliers and used without further purification. Reactions were performed under a nitrogen atmosphere. Melting point was determined using a Kofler camera Bock monoscope M (Lisboa, Portugal). Thin layer chromatography was performed using silica gel 60 F_254_ plates (Merck, Darmstadt, Germany) and visualized by ultraviolet (UV) light. For flash column chromatography high purity grade Merck silica gel (200–400 mesh) was used. ^1^H- and ^13^C-NMR spectra were recorded on a Fourier 300 Avance instrument (Bruker, Fällanden, Zurich, Switzerland) at 300 MHz (^1^H-NMR) and at 75 MHz (^13^C-NMR). ^1^H- and ^13^C-NMR chemical shifts are reported in parts per million (ppm, δ) referenced to the solvent used. Proton coupling constants (*J*) are expressed in hertz (Hz). Multiplicities are given as: s (singlet), dd (doublet of doublet), and m (multiplet). The specific rotation value was measured at r.t. on a 241 MC polarimeter (PerkinElmer, Waltham, MA, USA; New University of Lisbon, PT). Compound SLMP53-2 showed purity ≥90% by LC-MS, performed in an Alliance 2695 HPLC system (Waters, Dublin, Ireland) equipped with a Waters SunFire C18 column (100 × 2.1 mm; 5 µM) at 35 °C, using as mobile phase a gradient from 95% solution A (Milli-Q water containing 0.5% formic acid (v/v)) to 95% solution B (acetonitrile), and employing a photodiode array detector to scan wavelength absorption from 210 to 600 nm; MS experiments were performed on Micromass^®^ Quattro Micro triple quadrupole (Waters^®^, Dublin, Ireland) with an electrospray in positive ion mode (ESI+), ion source at 120 °C, capillary voltage of 3.0 kV and source voltage of 30 V, at the Liquid Chromatography and Mass Spectrometry Laboratory, Faculty of Pharmacy, University of Lisbon.

#### 4.2.2. Chemical Synthesis of SLMP53-2

Compound **1** was prepared from (*S*)-tryptophanol (0.16 g, 0.84 mmol, 1.0 equivalent) and 2-benzoyl-benzoic acid (0.21 g, 0.93 mmol, 1.1 equivalent) in 10 mL of toluene, according to the protocol described in reference [42]. To a stirred solution of compound **1** (0.10 g, 0.26 mmol) in anhydrous DMF (3 mL) at 0 °C, and under inert atmosphere of nitrogen, 0.014 g of NaH (0.58 mmol, 2.2 equivalent, 95% anhydrous reagent) was added. After stirring for 30 min, 0.025 mL of methyl iodide (0.39 mmol, 1.5 equivalent) was added and the reaction was slowly allowed to warm to room temperature for 1 h. Ethyl acetate (10 mL) was added, and the organic phase washed with water (6 × 10 mL), with an aqueous saturated solution of NaHCO_3_ and then with a brine solution. The organic phase was dried with Na_2_SO_4_ and concentrated. After flash chromatography (ethyl acetate/*n*-hexane 1:1) and recrystallization from EtOAc, SLMP53-2 was obtained as a white crystalline solid (0.096 g, 89.3%); mp: 134–136 °C; [α]^20^_D_ = + 84.5 (*c* = 0.37, CH_2_Cl_2_). ^1^H-NMR (CDCl_3_) δ 7.84–7.77 (m, 1H, ArH), 7.67–7.59 (m, 2H, ArH), 7.53–7.45 (m, 3H, ArH), 7.42–7.35 (m, 3H, ArH), 7.22 (m, 3H, ArH), 7.12–7.05 (m, 1H, ArH), 6.96 (s, 1H, ArH), 4.69 (m, 1H, H-3), 4.46 (dd, J = 8.6, 7.6 Hz, 1H, H-2), 3.98 (dd, J = 8.6, 6.8 Hz, 1H, H-2), 3.72 (s, 3H, NCH_3_), 3.21 (dd, J = 14.7, 5.5 Hz, 1H, CH_2_-indole), 2.65 (dd, J = 14.7, 9.3 Hz, 1H, CH_2_-indole); ^13^C-NMR (CDCl_3_) δ 174.72 (C = O), 147.29 (Cq), 138.98 (Cq), 136.98 (Cq), 133.38 (ArCH), 131.22 (Cq), 130.20 (ArCH), 128.87 (ArCH), 128.76 (ArCH), 128.01 (Cq), 127.01 (CH-indole), 125.92 (ArCH), 124.48 (ArCH), 123.56 (ArCH), 121.75 (ArCH), 119.01 (ArCH), 110.29 (Cq), 109.26 (ArCH), 101.06 (C-9b), 76.43 (CH-2), 55.91 (CH-3), 32.77 (N-CH_3_), 30.15 (CH_2_-indole); MS (ESI) m/z calcd for C_26_H_22_N_2_O_2_: 394, found 395 [M + H]^+^. 

### 4.3. Human Cell Culture Conditions

Human non-small cell lung carcinoma NCI-H1299, breast ductal carcinoma HCC1419, and non-tumorigenic foreskin fibroblast HFF-1 cell lines were purchased from ATCC (Rockville, MD, USA). The human HCC HuH-7 cell line was purchased from the JCRB cell bank (Osaka, Japan). The p53 knock-out (KO) HuH-7 cell line was generated using CRISPR editing [43]. Tumor cells were cultured in RPMI-1640 medium with UltraGlutamine (Lonza, VWR, Basel, Switzerland) with 10% FBS (Gibco, Alfagene, Lisboa, Portugal). HFF-1 cells were cultured in DMEM:F12 (Lonza) with 10% FBS. Cells were maintained in a humidified incubator at 37 °C with 5% CO_2_. Routine testing for Mycoplasma was performed using the MycoAlert™ PLUS detection kit (Lonza).

### 4.4. Transfection, Cell Proliferation, and Combination Therapy Assays

#### 4.4.1. Construction of Mammalian Expression Vectors

The full-length coding sequences of mutp53 were PCR-amplified from pLLS89 yeast vectors (kindly provided by Dr Gilberto Fronza, from IST Istituto Nazionale per la Ricerca sul Cancro, Italy) with Vent DNA polymerase (New England Biolabs, Werfen, Porto, Portugal), using the primers pair 5′ GGG GTA CCA TGG AGG AGC CGC AGT CAG 3′ and 5′ CCG CTC GAG TCA GTC TGA GTC AGG CCC TTC 3′, where the restriction sites for KpnI/XhoI (in bold) were included, respectively. The PCR products and the mammalian expression vector pcDNA3 (Invitrogen, Alfagene, Lisbon, Portugal) were digested with KpnI/XhoI (New England Biolabs, Werfen, Carnaxide, Portugal), purified from agarose gel, and ligated with T4 DNA ligase (Promega, VWR, Carnaxide, Portugal), originating the expression vectors pcDNA3-mutp53. These constructs were propagated in NZY5α *Escherichia coli* cells (NZYTech, Lisbon, Portugal). The sequence of each mutp53 in the constructed vectors was confirmed by sequencing (Eurofins GATC Biotech, Konstanz, Germany) with specific pcDNA3 primers. pcDNA3-mutp53 vectors were extracted using the PureYield™ Plasmid Miniprep System kit (Promega, VWR, Carnaxide, Portugal).

#### 4.4.2. Transfection

For ectopic mutp53 expression, p53-null NCI-H1299 cells were transfected with the previously obtained pCDNA3 plasmid encoding mutp53-R175H, -Y220C, -G245S, or -R280K, or empty pCDNA3. For Hsp70 knockdown, HuH-7 cells were transfected with ON-TARGETplus Human HSPA1A (3303) siRNA SMARTpool or nonspecific siRNAs (Non-targeting Pool) (Dharmacon, Bioportugal, Porto, Portugal). Cells in suspension were transfected using the ScreenFectA reagent and seeded immediately [44].

#### 4.4.3. SRB Assay

5.0 × 10^3^ (HuH-7, NCI-H1299) or 1.0 × 10^4^ (HCC1419, HFF-1) cells/well were seeded in 96-well plates and allowed to adhere overnight, followed by treatment with serial dilutions of compounds. Cell proliferation was measured as described [45]. IC_50_ values were determined using the GraphPad Prism software v7.0 (La Jolla, CA, USA). In combination therapy assays, combination index (CI) values were determined using the CompuSyn software v1.0 (ComboSyn, Inc., Paramus, NJ, USA) [46,47].

#### 4.4.4. Colony Formation Assay

2.0 × 10^3^ HuH-7 cells/well were seeded in 6-well plates and immediately treated with 0.88–28 µM SLMP53-2. Colonies were fixed and stained after 14 days, as described [47]. Colonies with more than 20 cells were counted.

### 4.5. Cell Cycle and Apoptosis Analysis

1.5 × 10^5^ HuH-7 cells/well were seeded in 6-well plates and allowed to adhere overnight, followed by treatment with 14 or 28 µM SLMP53-2 or DMSO for 48 h (cell cycle) or 72 h (apoptosis). Cell cycle and apoptosis were analyzed as described [45], using the Accuri™ C6 flow cytometer and the BD Accuri C6 software (BD Biosciences, Enzifarma, Porto, Portugal). FlowJo v10.0.7 (Treestar, Ashland, OR, USA) was used for quantification of cell cycle phases.

### 4.6. Western Blot

1.5 × 10^5^ HuH-7 or NCI-H1299 cells/well were seeded in 6-well plates and allowed to adhere overnight, followed by treatment with 14 µM SLMP53-2. Sample preparation and western blot were performed as described [48]. Signal detection was carried out using the ECL Prime Amersham kit (GE Healthcare, VWR, Carnaxide, Portugal) and the ChemiDoc™ MP Imaging System (Bio-Rad Laboratories, Amadora, Portugal). Whole blot images (Figure S12) and blot quantifications (Table S7) are provided in supplementary material.

### 4.7. IP and co-IP

2 × 10^6^ HuH-7 cells were seeded in 75 cm^2^ flasks, allowed to adhere overnight, followed by 36 h treatment with 28 or 42 µM SLMP53-2 or DMSO. The Pierce Classic Magnetic IP/Co-IP Kit (Thermo Scientific, Dagma, Carcavelos, Portugal) was used according to manufacturer’s instructions. For IP, 1 µg/mL PAb1620 (wt/folded) or PAb240 (mut/unfolded) conformation-specific anti-p53 antibodies were used. For Co-IP, 1 µg/mL anti-p53 antibody was used. Hsp40/Hsp70/Hsp90, p53, and GAPDH (loading control) were detected by western blot, in immunoprecipitated fractions and whole cell lysates (input), as described above.

### 4.8. RNA Extraction and RT-qPCR

1.5 × 10^5^ HuH-7 cells/well were seeded in 6-well plates and allowed to adhere overnight, followed by 24 h treatment with 14 or 28 µM SLMP53-2 or DMSO.

#### 4.8.1. Gene Expression Analysis

Total RNA extraction and RT-qPCR were performed as described [49], using specific primers for each analyzed gene (Table S6) (Eurofins, MWG, Milan, Italy); GAPDH and B2M were used as reference genes.

#### 4.8.2. miRNA Analysis

Total RNA was extracted using TRIzol reagent (Invitrogen, Carlsbad, CA, USA) according to manufacturer’s instructions. RNA concentration and purity were measured in NanoDrop™ 1000 (Thermo Fisher Scientific, Waltham, MA, USA). 260/280 nm and 260/230 nm ratios ranged around 1.8–2.2. RNA integrity was assessed by gel electrophoresis. miRNA levels were evaluated using TaqMan miRNA assays (Applied Biosystems, Foster City, CA, USA). cDNA was synthesized (MyCycler Thermal Cycler, Bio-Rad) using RNA, TaqMan MicroRNA Reverse Transcription Kit (Applied Biosystems) and stem-loop Reverse Transcription primers (hsa-miR-34a-5p; Applied Biosystems). qPCR reactions were performed in CFX96 Touch™ Real-Time PCR Detection System (Bio-Rad) using cDNA, miR-34a or snRNA U6 TaqMan probes (Applied Biosystems) and SsoAdvanced™ Universal Probes Supermix (Bio-Rad, Hercules, CA, USA). snRNA U6 was used as reference gene. Relative expression levels were calculated using the quantification cycle (Cq) method, according to MIQE guidelines [50].

### 4.9. ChIP

1.5 × 10^6^ HuH-7 cells were seeded in 150 mm dishes and allowed to adhere overnight, followed by 24 h treatment with 28 µM SLMP53-2 or DMSO. ChIP protocol was performed as described [51]. p53 occupancy at the p21 promoter was measured by RT-qPCR (primers are listed in Table S6).

### 4.10. Microarray Experiments

2.0 × 10^6^ HuH-7 cells were seeded in 6-well plates and allowed to adhere overnight, followed by 24 h treatment with 28 or 42 µM SLMP53-2 or DMSO. Total RNA was extracted using Illustra™ RNAspin Mini (GE Healthcare, Chicago, IL, USA), purity and concentration were measured by Nanodrop and Agilent 2100 Bioanalyzer (Agilent Technologies, Santa Clara, CA, USA). Samples with RIN (RNA integrity number) ≥8 were used for the one-Color Microarray-Based Gene Expression Analysis protocol (Agilent Technologies, Santa Clara, CA, USA) and hybridized on Agilent-014850 4x44K Whole Human Genome Microarrays. Slides were scanned on the Agilent DNA Microarray Scanner (G2505C) using the Agilent HD_GX_1Color Profile of Agilent ScanControl v8.1.3 (Agilent Technologies, Santa Clara, CA, USA). Four biological replicates per treatment were prepared and analyzed. Microarray data have been deposited in Gene Expression Omnibus database (GSE124021). 

The scanned TIFF images were analyzed numerically and background-corrected using the Agilent Feature Extraction Software v10.7.7.1 (Agilent Technologies, Santa Clara, CA, USA), according to the Agilent GE1_107_Sep09 standard protocol. The output of Feature Extraction was analyzed with the R software environment for statistical computing (http://www.r-project.org/) and the Bioconductor packages (http://www.bioconductor.org/). The array Quality Metrics package was used to check the quality of the arrays. Low signal Agilent probes, identified by a repeated “not detected” flag across the majority of the arrays in every condition, were filtered out from the analysis. Signal intensities across arrays were background corrected (Edwards method) and normalized with the quantile normalization method. DEGs were determined using a double threshold based on: (1) the magnitude of the change (fold change greater than ± 2); (2) the statistical significance of the change, measured with a multiple-test-correction adjusted *p*-value less than 0.05 using limma package. The IPA (v2017) resource was used for enrichment analysis of the transcriptome DEGs lists. The IPA Ontologies are derived from manually curated collections of experimental data and are utilized to infer functional enrichment of different type of relationships. The significance of gene list over-representations was determined using a Fisher exact *p*-value threshold of 0.05 with multiple-test-correction. In particular, the bio-functions and the up-stream regulators analysis were used. The graphical representation of the IPA results was performed in R/Bioconductor environment.

### 4.11. Immunofluorescence

Immunofluorescence staining of HuH-7 cells was performed basically as described [52]. 

#### 4.11.1. XBP1 Staining

HuH-7 cells were seeded in 24-well plates at 1.5 × 10^5^ cells/well density and allowed to adhere overnight, followed by 24 h treatment with 28 µM SLMP53-2 or DMSO. After treatments, cells were fixed with 4% formaldehyde/PBS for 20 min at room temperature (RT), were blocked with BSA 5% and Triton 0.1% for 1h at RT, incubated with anti-XBP1 antibody overnight at 4 °C, followed by incubation with an Alexa Fluor 488-conjugated secondary antibody for 1 h at RT, and staining with Hoechst (ThermoFisher Scientific, Waltham, MA, USA; 1:10,000). Images were visualized with a Zeiss Axio Observer Z1 microscope (Carl Zeiss, Oberkochen, Germany) using Zeiss AxioVision v4.8.1. Nuclear signal was quantified using CellProfiler^TM^ software v3.1.5 (Broad Institute, Cambridge, MA, USA).

#### 4.11.2. PAb240/PAb1620 Staining

HuH-7 cells were seeded in chambered cell culture slides at 1 × 10^4^ cells/well density and allowed to adhere overnight, followed by 36 h treatment with 42 µM SLMP53-2 or DMSO. After treatment, the cell monolayer was fixed with 4% formaldehyde/PBS for 30 min at RT, permeabilized with 0.1% Triton for 20 min at 4 °C, blocked with 5% BSA for 1h at RT, incubated with PAb240, PAb1620, or DO-1 anti-p53 antibodies overnight at 4 °C, followed by incubation with Alexa Fluor 488-conjugated secondary antibody for 1h at RT, and staining with DAPI. Cells were photographed (Nikon DS-5Mc camera; Nikon Eclipse E400 fluorescence microscope; Nikon ACT-2U software, Izasa, Carnaxide, Portugal).

### 4.12. HSQC-NMR

The stabilized DBD of the mutp53-Y220C (T-p53C-Y220C) was expressed and purified as described [29]. For expression of its ^15^N-labeled form, M9 minimal medium with ^15^NH_4_Cl (1 g/L) as sole nitrogen source was used.

^1^H-^15^N HSQC spectra of ^15^N-labeled T-p53C-Y220C (70 μM) and different compound concentrations were recorded and analyzed as described [29]. Briefly, the spectra were recorded at 293 K on a Bruker Avance-800 spectrometer using a 5-mm inverse cryogenic probe. Compound samples were mixed with protein immediately before the NMR measurement. Analysis of spectra was performed using Sparky 3.11430 (San Francisco, CA, USA) and Bruker Topspin 2.0 software (Coventry, UK).

### 4.13. In Vivo Anti-Tumor and Toxicity Assays

All animal experiments were conducted following the EU Directive 2010/63/EU and National Authorities. The study was approved by the local Animal Welfare Body (Ref. ORBEA-5-2016). Swiss nude mice (CharlesRiver Laboratories, Barcelona, Spain) were housed under pathogen-free conditions in ventilated cages. 7.5 × 10^6^ HuH-7 cells (in PBS/Matrigel 1:1; Corning, Enzifarma, Porto, Portugal) were subcutaneously inoculated in the dorsal flank of female Swiss nude mice (five animals/group). Tumors were routinely measured using a caliper for calculation of tumor volume by the formula (a × b^2^)/2 (a and b represent the longest and shortest tumor axes, respectively). Twice-weekly intraperitoneal injections of 50 mg/kg SLMP53-2 or vehicle were started for tumors with approximately 100 mm^3^ (5 days after the grafts). Five administrations were performed with continuous monitoring of tumor volume, animal weight, and signs of morbidity. At the end of treatment, animals were sacrificed by cervical dislocation. For toxicity assays, female Wistar rats were treated with 50 mg/kg SLMP53-2 or vehicle (DMSO) by intraperitoneal injection, twice a week. After five administrations, blood samples were collected for toxicological analysis. Each experimental group was composed of four animals.

### 4.14. Immunohistochemical (IHC) Analysis

IHC of xenograft tumor tissue was performed as described [49]. Evaluation of DAB intensity and quantification were performed using ImageJ v1.8.0. (Madison, WI, USA). 

### 4.15. Statistical Analysis

Data were statistically analyzed using the GraphPad Prism software v7.0 (GraphPad Inc., La Jolla, CA, USA). Appropriate statistical tests were applied to each dataset; *p* < 0.05 was considered statistically significant.

## 5. Conclusions

The present work brings a new mutp53-reactivating compound with a distinct mechanism of action from those currently reported. Notably, SLMP53-2 may also represent a new hope in cancer treatment, either alone or in combination therapy, particularly against HCC. In addition, it represents a lead compound and therefore a valuable starting material for new optimized derivatives. In fact, this work highlights the tryptophanol-derived oxazoloisoindolinones as a promising chemical family for the development of more effective anticancer therapeutic options by targeting a larger set of mutp53 forms.

## 6. Patents

The compound SLMP53-2 is protected under an international patent (European patent EP3013833 and US patent 20160347765).

## Supplementary Materials

The following are available online at https://www.mdpi.com/2072-6694/11/8/1151/s1, Figure S1: Clustering analysis of the microarray data, Figure S2: Microarray data analysis related to Figure 2, Figure S3: Connectivity map results, Figure S4: Representative images of XBP1 immunofluorescence in HuH-7 cells treated with 28 µM SLMP53-2 or DMSO for 24 h, Figure S5: Protein levels of p53 target genes in HCC1419 cells, after 24 h (KILLER) or 48 h (MDM2, p21, survivin, and VEGF) treatment with 14 µM SLMP53-2, Figure S6: Representative images of p53 immunofluorescence staining of HuH-7 cells treated with 42 µM SLMP53-2 or DMSO for 36 h, Figure S7: Overlay of 1H/15N-HSQC NMR spectra of T-p53C-Y220C with varying concentrations of SLMP53-2, showing no significant chemical shift, Figure S8: Western blot analysis of Hsp70 protein levels in HuH7 cells transfected with sipHsp70 or siCTRL, Figure S9: ^1^H NMR and ^13^C NMR data for SLMP53-2, Figure S10: Concentration-response curves for SLMP53-2 in (A) HCT116 p53^+/+^, HCT116 p53^−/−^, and (B) HepG2 cells, analyzed by SRB assay after 48h treatment with 3.12–50 µM SLMP53-2, Figure S11: Co-immunoprecipitation of Hsp70 with p53 in HuH-7 cells treated with 34 and 68 µM SLMP53-1 or DMSO for 36 h, using anti-p53 antibody (DO-1), followed by immunoblotting with anti-Hsp70and anti-p53 antibodies, Figure S12: Whole blots. Table S1: Gene expression data (see Table S1.xlsx file), Table S2: Ontology, Pathways and Upstream Regulators analyses by Ingenuity Pathway and Metascape (see Table S2.xlsx file), Table S3: Connectivity Map results by Ingenuity Pathway analysis of the microarray data (see Table S3.xlsx file), Table S4: Biochemical and hematological data of SLMP53-2 in Wistar rats, Table S5: List of antibodies used in western blot (WB), immunofluorescence (IF), immunohistochemistry, and immunoprecipitation, Table S6: List of primers, Table S7: Quantification of western blots.
