# Supplementary material for: SLMP53-2 Restores Wild-Type-Like Function to Mutant p53 through Hsp70: Promising Activity in Hepatocellular Carcinoma
Source: Cancers (Basel). 2019 Aug 10;11(8):1151. doi: 10.3390/cancers11081151 (PMC6721528; doi:10.3390/cancers11081151)
Supplement: Supplementary file 1 [file cancers-11-01151-s001.pdf]

Supplementary Material: SLMP53-2 Restores Wild-Type-Like Function to Mutant p53 through Hsp70: Promising Activity in Hepatocellular Carcinoma

Sara Gomes, Bartolomeo Bosco, Joana B. Loureiro, Helena Ramos, Liliana Raimundo, Joana Soares, Nair Nazareth, Valentina Barcherini, Lucília Domingues, Carla Oliveira, Alessandra Bisio, Silvano Piazza, Matthias R. Bauer, João P. Brás, Maria Inês Almeida, Célia Gomes, Flávio Reis, Alan R. Fersht, Alberto Inga, Maria M. M. Santos, Lucília Saraiva

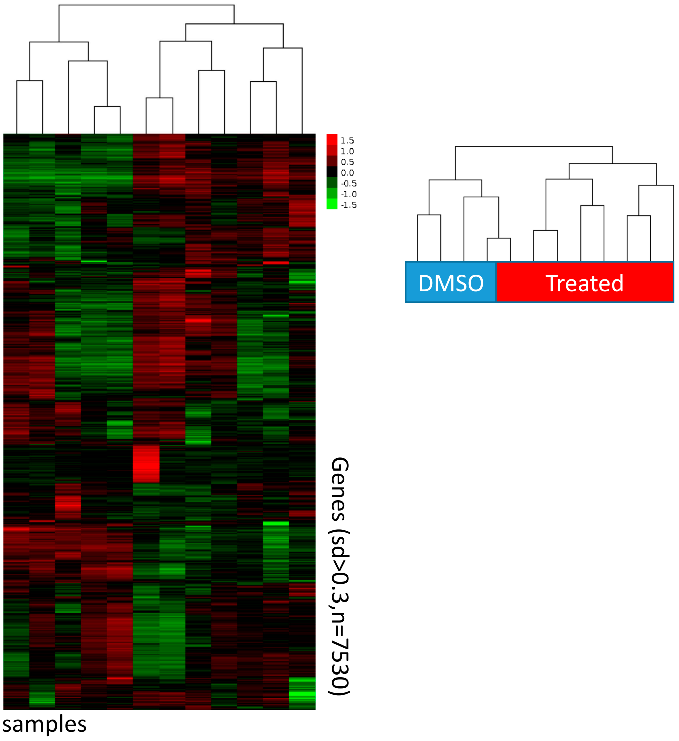

Figure S1. Clustering analysis of the microarray data. The microarray experiment was performed in quadruplicate for 2xIC50 or 3xIC50 SLMP53-2 and control (DMSO). The overall result of the clustering analysis performed expression matrix of the genes with standard deviation higher than 0.3 (n=7530) indicates high level of reproducibility among the replicates. The microarray data has been deposited in GEO (GSE124021).

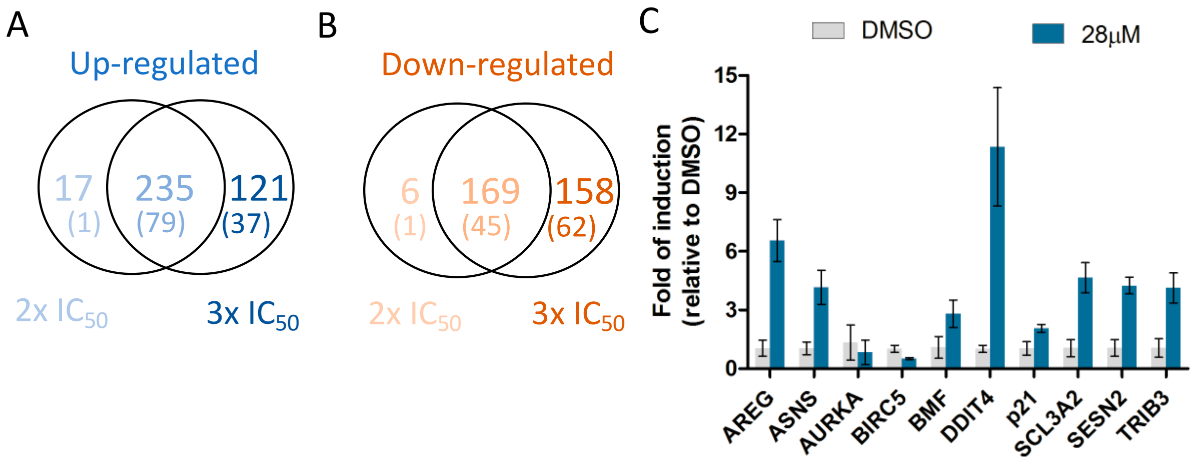

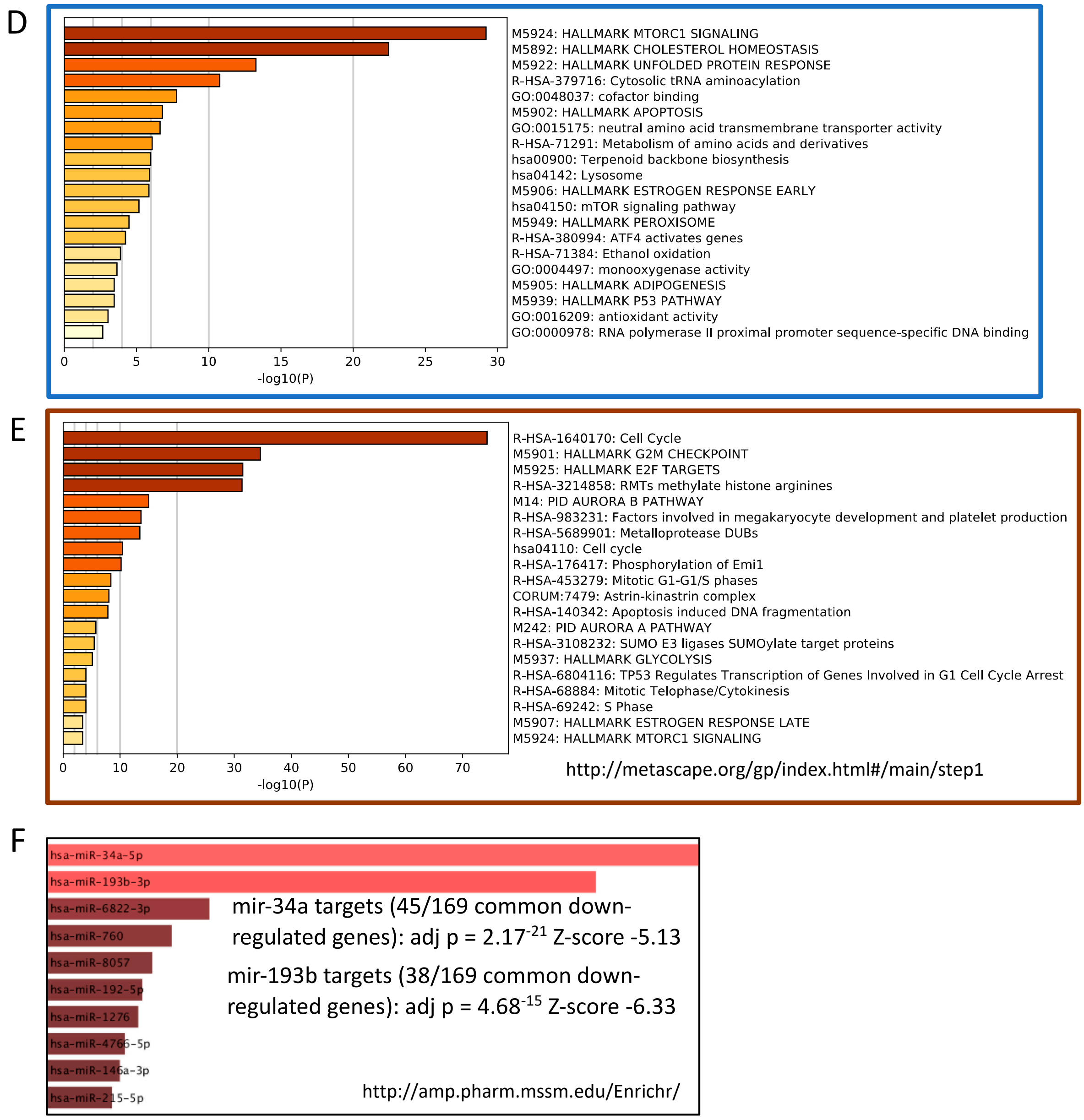

Figure S2. Microarray data analysis related to Figure 2. Venn Diagram presenting the number of DEGs (adj p value < 0.05) for 2 × IC_{50} or 3 × IC_{50} SLMP53-2 and the overlap. Both for Up-regulated (**A**) and Down-regulated (**B**) genes there is a dose dependency on the number of DEGs with a large common core of genes. Besides the p value cut-off, a $\log_2$ fold change greater or equal to 0.6 or less than −0.6 was used. Number in parenthesis are relative to a more stringent cut-off ($\log_2$ fold change cut off >1 or <−1). (**C**) qPCR validation of the responsiveness of 10 DEGs for 2 × IC50 SLMP53-2. Two of the ten genes (*AURKA* and *BIRC5*) were repressed by SLMP53-2 according to the microarray results. In all cases except *AURKA*, for which the difference is consistent but not significant, SLMP53-2 led to significant modulation of the genes ($p < 0.05$, t-test). The highest fold change was seen for *DDIT4* that along with *SESN2* and *TRIB3* were included because of their involvement in the unfolded protein response. Presented are the average fold of induction relative to the DMSO treatment and the standard deviation of three replicates. B2M was used as reference genes. (**D**,**E**) Metascape was used to perform Gene Ontology analysis and comparison of enriched pathways and molecular functions derived from the lists of DEGs in common between the two treatments. (**D**) Modulation of mTORC1 signaling, cholesterol homeostasis and unfolded protein response are the most significant enriched features from the up-regulated gene group, consistent with the results obtained by Ingenuity Pathway (Figure S2). (**E**) Cell cycle, proliferation and cell division pathways are strongly enriched considering the repressed,

differentially expressed genes. (**F**) SLMP53-2 repressed genes are enriched for mir-34a and mir-193b targets according to Enrichr. Presented are the results from miRTarBase-2017 starting from the list of 169 down-regulated, genes that are downregulated in the treatment with both SLMP53-2. The number of targets for each of the two microRNAs and the statistical analysis is provided.

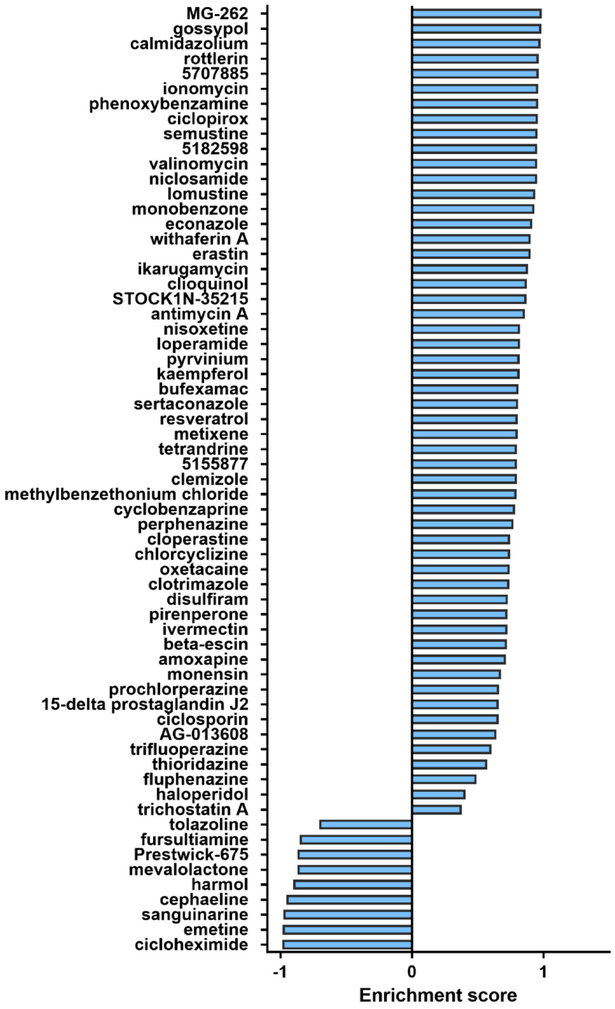

Figure S3. Connectivity map results. Summary graph of the connectivity map results obtained using the gene expression results from the HuH-7 cells treated with 2xIC$_{50}$ SLMP53-2. Results were filtered for *p* value and are ordered for decreasing enrichment score. As expected given the strong cell cycle arrest and apoptosis phenotype induced by SLMP53-2, high enrichment score was observed with several drugs, although specificity was generally low. Interestingly, among the top scorer are molecules involved in autophagy and proteasome functions. Further, cytotoxic chemotherapeutics are not present in the top scoring molecules, consistent with the results data SLMP53-2 is not activating an overt DNA damage response (Figure 2D).

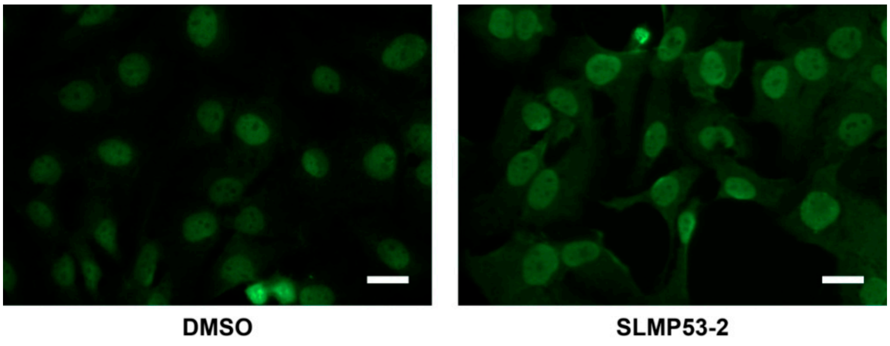

Figure S4. Representative images of XBP1 immunofluorescence in HuH-7 cells treated with 28 μM SLMP53-2 or DMSO for 24 h (scale bar = 10 μm).

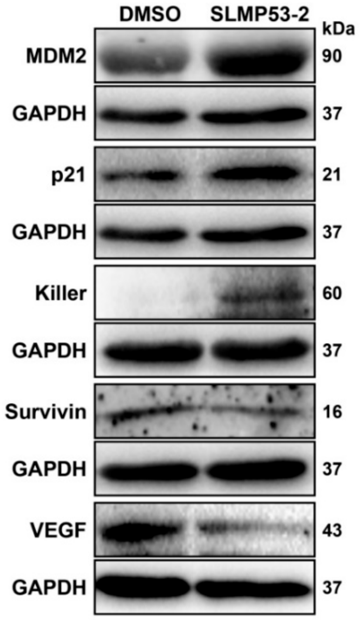

Figure S5. Protein levels of p53 target genes in HCC1419 cells, after 24 h (KILLER) or 48 h (MDM2, p21, survivin, and VEGF) treatment with 14 μM SLMP53-2. Immunoblots represent one of three independent experiments; GAPDH was used as a loading control.

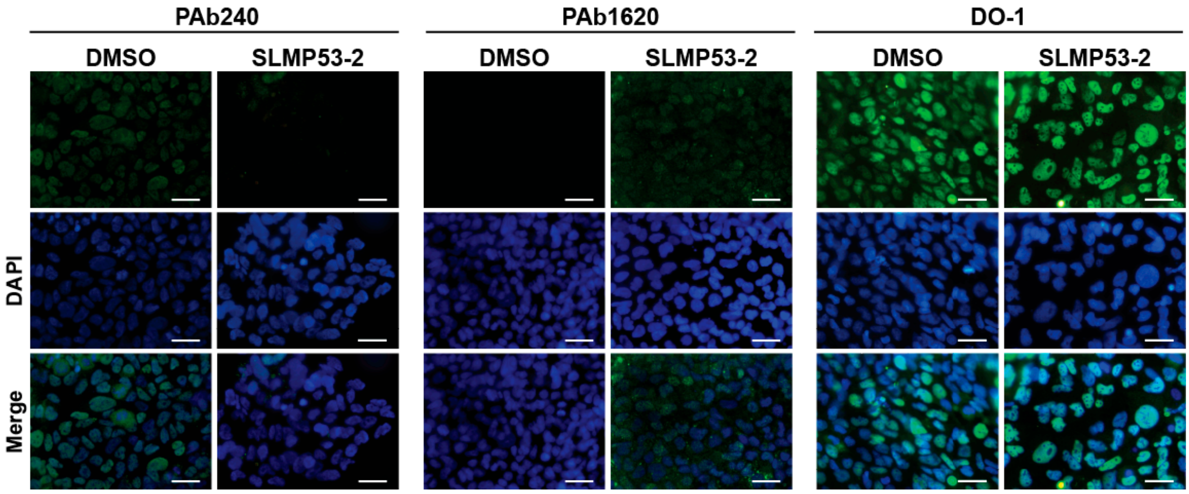

Figure S6. Representative images of p53 immunofluorescence staining of HuH-7 cells treated with 42 μM SLMP53-2 or DMSO for 36 h. Cells were labelled with conformation-specific antibodies PAb240 (unfolded/mutant) and PAb1620 (folded/wild-type), or with DO-1 (total p53) (scale bar = 20 μM).

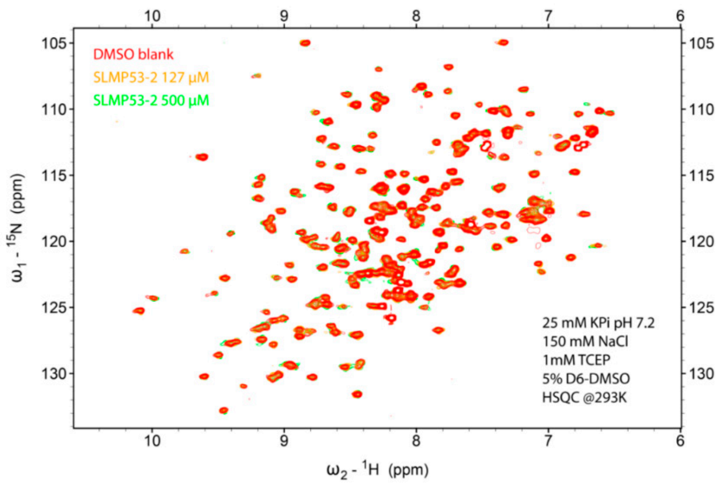

Figure S7. Overlay of $^{1}H/^{15}N$-HSQC NMR spectra of T-p53C-Y220C with varying concentrations of SLMP53-2, showing no significant chemical shift.

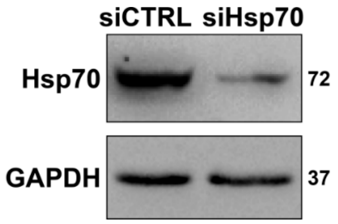

Figure S8. Western blot analysis of Hsp70 protein levels in HuH7 cells transfected with sipHsp70 or siCTRL.

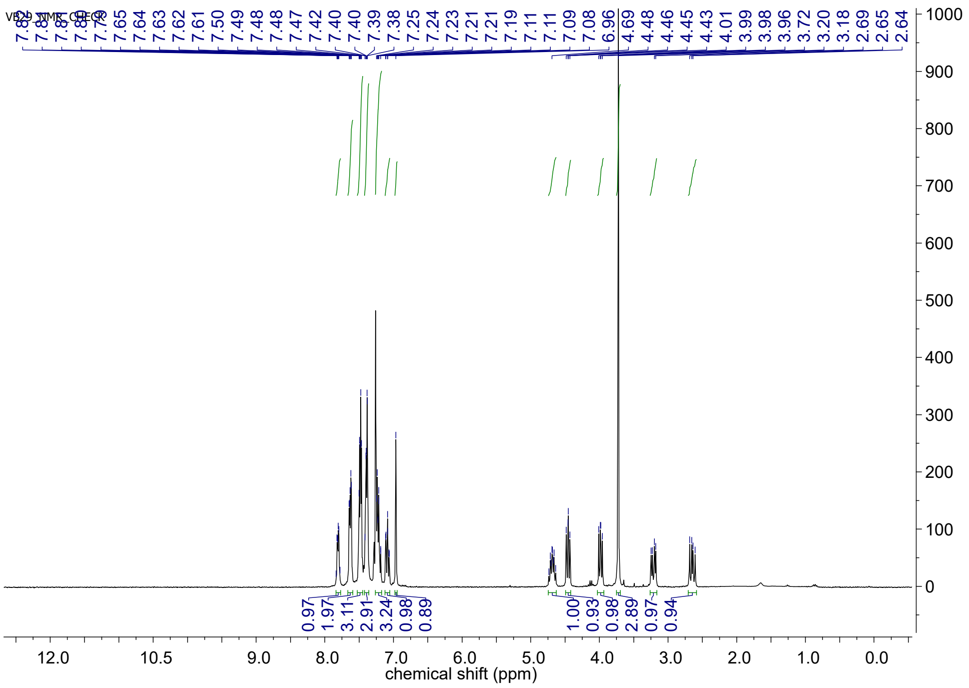

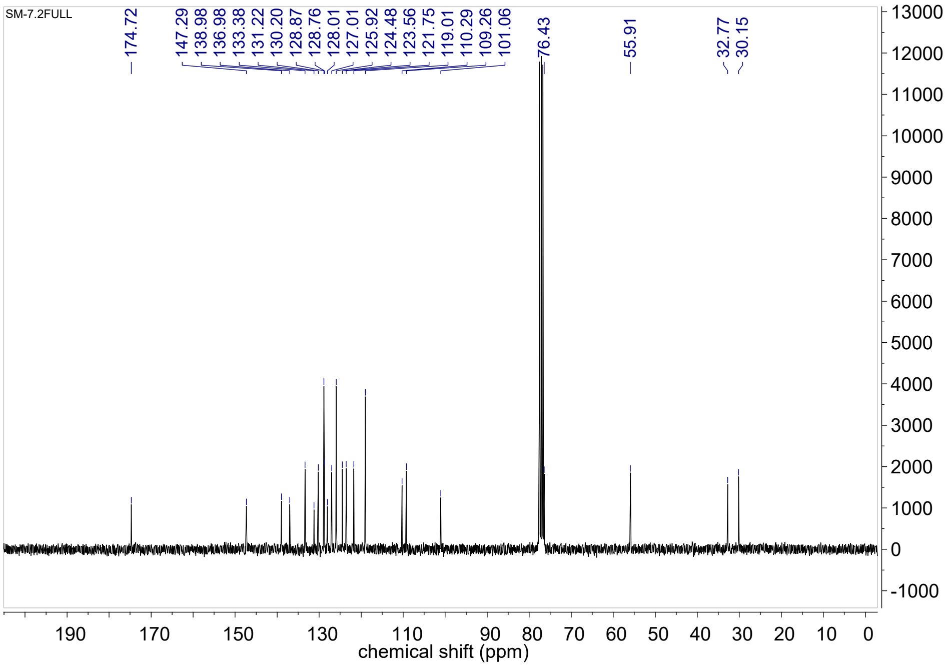

Figure S9. ^{1}H NMR and ^{13}C NMR data for SLMP53-2.

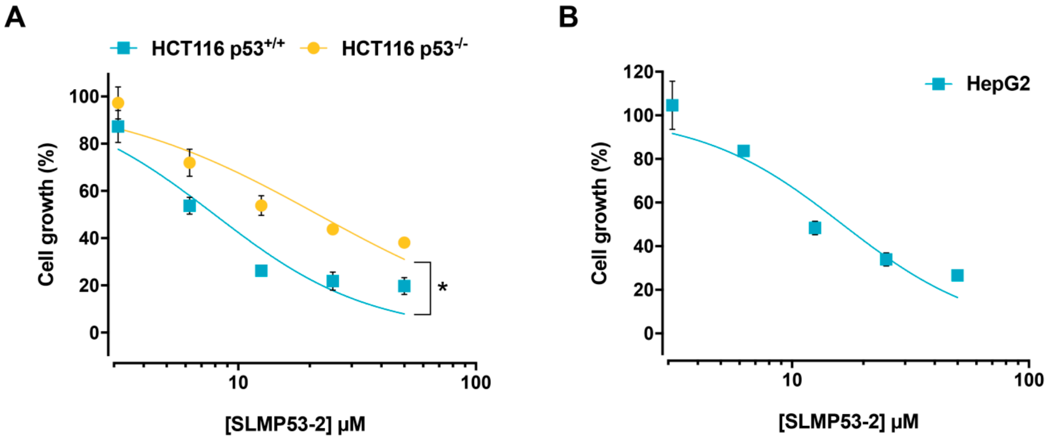

Figure S10. Concentration-response curves for SLMP53-2 in (**A**) HCT116 $p53^{+/+}$, HCT116 $p53^{-/-}$, and (**B**) HepG2 cells, analyzed by SRB assay after 48h treatment with 3.12–50 μM SLMP53-2. Data are mean ± SEM ($n = 3$); * $p < 0.05$, extra sum-of-squares F test.

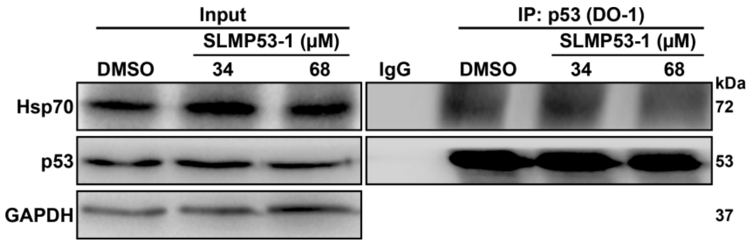

Figure S11. Co-immunoprecipitation of Hsp70 with p53 in HuH-7 cells treated with 34 and 68 μM SLMP53-1 or DMSO for 36 h, using anti-p53 antibody (DO-1), followed by immunoblotting with anti-Hsp70and anti-p53 antibodies; whole cell lysate (input); immunoblots represent one of three independent experiments; GAPDH was used as a loading control.

Figure 1

p53 **GAPDH**

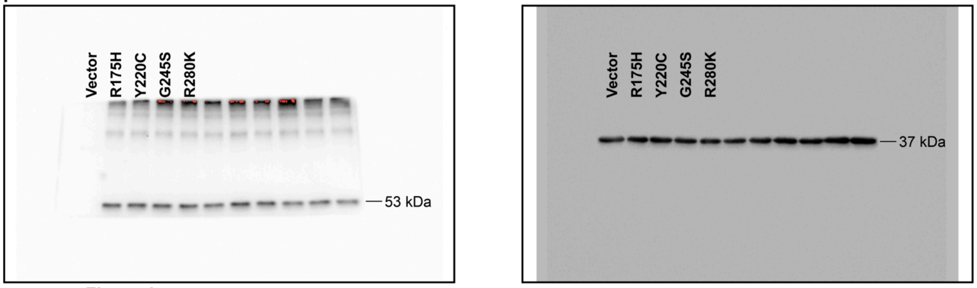

Figure 3

eIF2α **p-eIF2α**

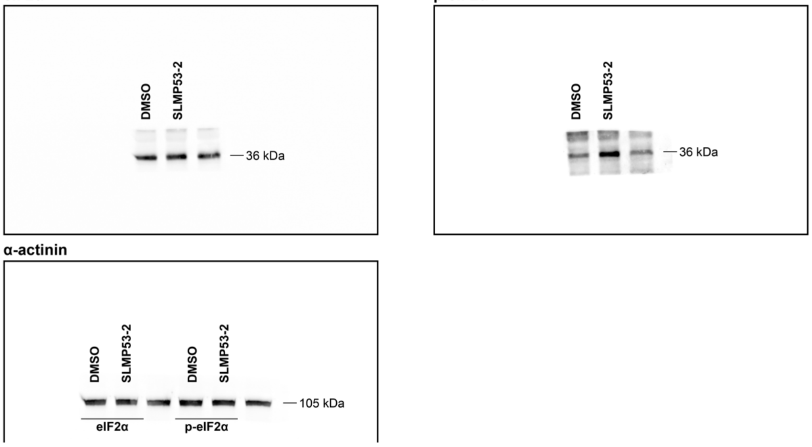

Figure 4

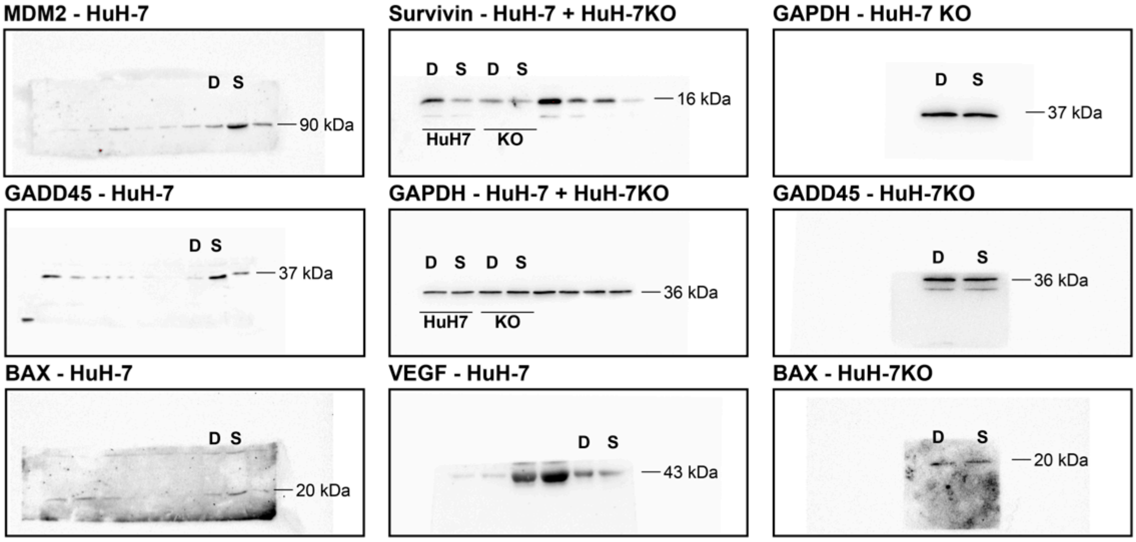

GAPDH - HuH-7 **GAPDH - HuH-7** **GAPDH - HuH-7KO**

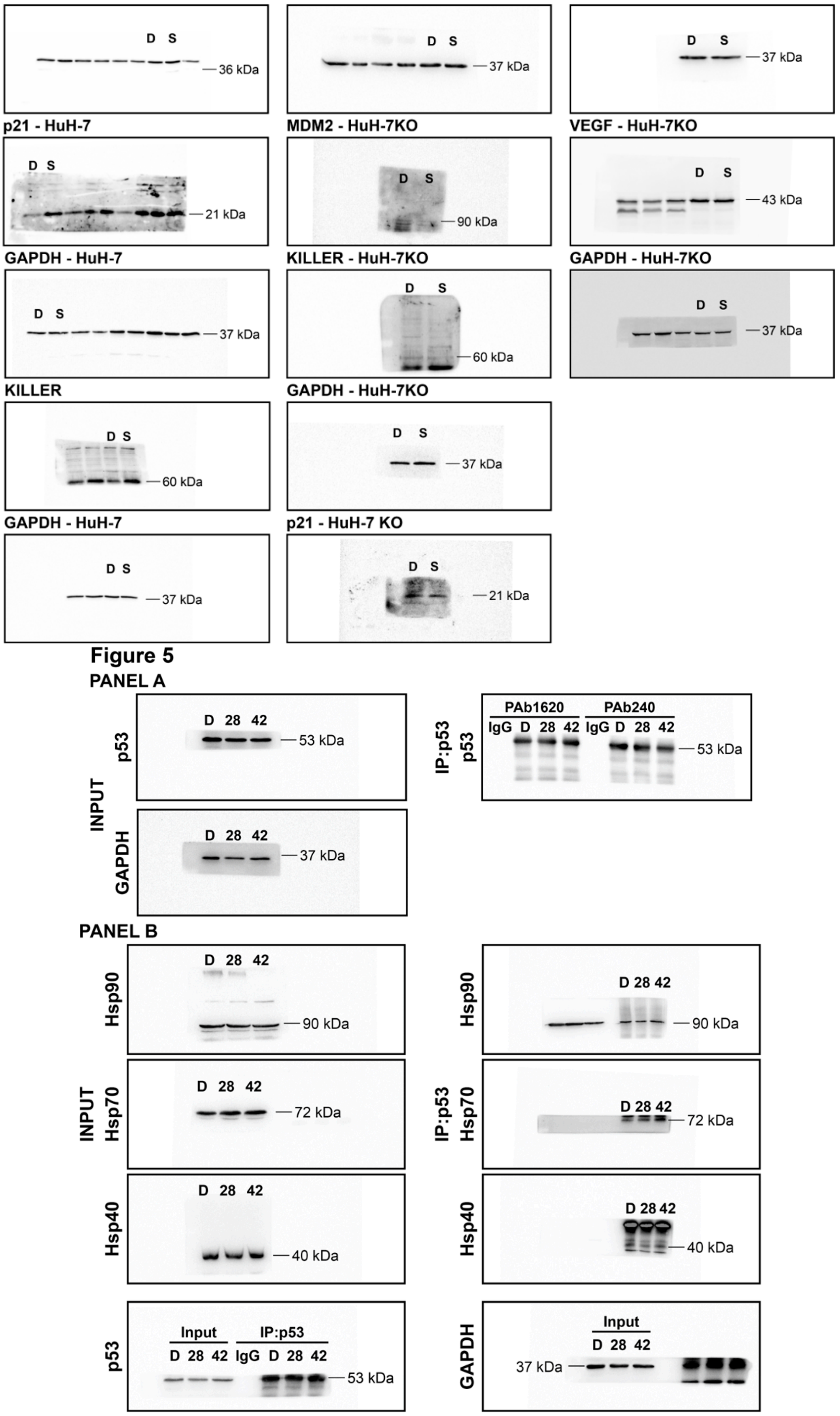

Figure S12. Whole blots.

Table S1. Gene expression data (see Table S1.xlsx file). The two sheets contain the gene expression data resulting from the treatment with 2xIC_{50} or 3xIC_{50} (28 or 42μM) SLMP53-2. Gene Name, Systematic Name, Description, Log2 Fold Change, Average expression signal and statistical analysis are presented.

Table S2. Ontology, Pathways and Upstream Regulators analyses by Ingenuity Pathway and Metascape (see Table S2.xlsx file). Three sheets are included for each treatment dose (2xIC_{50} or 3xIC_{50} SLMP53-2) presenting the results of Canonical Pathways, Upstream Regulator, Disease and Biofunctions, according to Ingenuity Pathway (see Figure S2). Two additional sheets contain the Gene Ontology results obtained with Metascape (metascape.org/) (see Figure S4).

Table S3. Connectivity Map results by Ingenuity Pathway analysis of the microarray data (see Table S3.xlsx file). "Cmap molecule name" data obtained comparing the gene expression results of 2xIC_{50} or 3xIC_{50} SLMP53-2. Tables are filtered based on *p* value and rank. Results obtained with the 2xIC_{50} SLMP53-2 are summarized in Figure S4.

Table S4. Biochemical and haematological data of SLMP53-2 in Wistar rats.

Parameter	Control	Treated
**Biochemical data**		
Blood Glucose (mg/dL)	165.83 ± 8.91	193.33 ± 19.63
Urea (mg/dL)	18.38 ± 0.45	19.07 ± 0.62
Creatinine (mg/dL)	0.32 ± 0.01	0.32 ± 0.01
Uric Acid (mg/dL)	2.8 ± 0.33	1.9 ± 0.15
Total protein (g/dL)	6.68 ± 0.24	6.43 ± 0.03
Albumin (g/dL)	3.25 ± 0.08	3.4 ± 0.06
Sodium (mmol/L)	146.83 ± 0.83	148.33 ± 1.33
Potassium (mmol/L)	5.98 ± 0.23	5.83 ± 0.67
Osmolality (mOSM/Kg)	296.6 ± 1.97	302.67 ± 1.76
Phosphorous (mg/dL)	7.83 ± 0.47	8.43 ± 0.17
ALT (U/L)	38.67 ± 2.5	34.33 ± 3.84
AST (U/L)	73.83 ± 8.68	67.67 ± 5.46
Total Cholesterol (mg/dL)	65.33 ± 7.44	68.67 ± 2.96
Cholesterol-HDL (mg/dL)	42.33 ± 4.78	44 ± 2.08
Cholesterol-LDL (mg/dL)	20.83 ± 2.79	19.67 ± 1.45
Triglycerides (mg/dL)	153.33 ± 27.72	183 ± 10.07
Atherogenic index	1.53 ± 0.04	1.57 ± 0.03
**Hematological data**		
WBC (×10^3/μL)	1.78 ± 0.42	2.33 ± 0.87
RBC (×10^6/μL)	8.5 ± 0.32	8.14 ± 0.49
HGB (g/dL)	14.72 ± 0.55	14.9 ± 0.15
HCT (%)	44.07 ± 1.69	44.7 ± 0.61
MCV (fL)	51.85 ± 0.58	55.23 ± 3.47
MCH (pg)	17.3 ± 0.21	18.4 ± 1.11
MCHC (g/dL)	33.38 ± 0.3	33.33 ± 0.15
RDW (%)	14.73 ± 0.31	13.3 ± 0.55*
PLT (×10^3/μL)	552.98 ± 152.92	781.67 ± 29.04
MPV (fL)	7.92 ± 0.02	6.23 ± 0.83*
RET (%)	3.09 ± 0.31	3.66 ± 0.16
IRF	0.7 ± 0.03	0.71 ± 0.01
Lymphocytes (%)	77.3 ± 1.03	81 ± 3.91
Lymphocytes (×10^3/μL))	1.4 ± 0.32	1.83 ± 0.61

Data from blood samples were analysed for saline (control), and 50mg/kg SLMP53-2 (treated) rat groups, after five intraperitoneal administrations (twice a week). Results are shown as mean±SEM (n=5; values significantly different from control: * $p < 0.05$; unpaired Student's *t*-test). ALT, alanine aminotransferase; AST, aspartate aminotransferase; HCT, haematocrit; HGB, Haemoglobin concentration; IRF, immature reticulocyte fraction; MCH, mean corpuscular haemoglobin; MCHC,

mean corpuscular haemoglobin concentration; MCV, mean corpuscular volume; MPV, mean platelet volume; PLT, platelet; RBC, red blood cell count; RDW, red cell distribution width; RET, reticulocytes; WBC, white blood cells.

Table S5. List of antibodies used in western blot (WB), immunofluorescence (IF), immunohistochemistry, and immunoprecipitation.

Antigen	Final Dilution	Supplier
Primary antibodies		
**α-actinin**<br>Mouse monoclonal	1:6000	Santa Cruz Biotechnology<br>**Cat# sc-17829**
**γH2AX**<br>Rabbit polyclonal	1:10000	Abcam<br>**Cat# ab2893**
**Bax** (6A7)<br>Mouse monoclonal	1:100	Thermo Scientific<br>**Cat# MA5-14003**
**eIF2α**<br>Rabbit polyclonal	1:500	Abcam<br>**Cat# ab26197**
**p-eIF2α**<br>Rabbit polyclonal	1:500	Abcam<br>**Cat# ab32157**
**GADD45**<br>Rabbit polyclonal	1:500	Millipore<br>**Cat# ABE2696**
**GAPDH** (6C5)<br>Mouse monoclonal	1:10000	Santa Cruz Biotechnology<br>**Cat# sc-32233**
**Hsp40** (B-3)<br>Mouse monoclonal	1:200	Santa Cruz Biotechnology<br>**Cat# sc-398766**
**Hsp70**<br>Rabbit polyclonal	1:1000	Sigma-Aldrich<br>**Cat# SAB2702387**
**Hsp90** (F-8)<br>Mouse monoclonal	1:500	Santa Cruz Biotechnology<br>**Cat# sc-13119**
**Killer**<br>Rabbit polyclonal	1:500	Thermo Scientific<br>**Cat# PA5-19895**
**Ki67** (SP6)<br>Rabbit monoclonal	1:500	Thermo Scientific<br>**Cat# MA5-14520**
**MDM2** (SMP14)<br>Mouse monoclonal	1:100	Santa Cruz Biotechnology<br>**Cat# sc-965**
**p21** (C-19)<br>Rabbit polyclonal	1:100	Santa Cruz Biotechnology<br>**Cat# sc-397**
**p53** (DO-1)<br>Mouse monoclonal	1:5000 (WB)<br>1:500 (IF)	Santa Cruz Biotechnology<br>**Cat# sc-126**
**p53** (PAb1620; Ab-5)<br>Mouse monoclonal	1:100	Millipore<br>**Cat# OP33**
**p53** (PAb240; Ab-3)<br>Mouse monoclonal	1:200	Millipore<br>**Cat# OP29**
**Survivin** (EP2880Y)<br>Rabbit monoclonal	1:15000	Abcam<br>**Cat# ab76424**
**VEGF**<br>Mouse monoclonal	1:200	Thermo Scientific<br>**Cat# MA1-16629**
**XBP1**<br>Mouse monoclonal	1:250	Santa Cruz Biotechnology<br>**Cat# sc-8015**
Secondary antibodies		
**Anti-mouse**<br>HRP-conjugated	1:5000	Santa Cruz Biotechnology<br>**Cat# sc-2005**
**Anti-rabbit**<br>HRP-conjugated	1:5000	Santa Cruz Biotechnology<br>**Cat# sc-2006**
**Anti-mouse**<br>Alexa Fluor 488-conjugated	1:1000	ThermoFisher Scientific<br>**Cat#A-11001**

Immunoprecipitation		
**p53** (DO-1)<br>Mouse monoclonal	1µg/mL	Santa Cruz Biotechnology<br>**Cat# sc-126**
**p53** (PAb1620; Ab-5)<br>Mouse monoclonal	1µg/mL	Millipore<br>**Cat# OP33**
**p53** (PAb240; Ab-3)<br>Mouse monoclonal	1µg/mL	Millipore<br>**Cat# OP29**

Table S6. List of primers.

Primer	Sequence (5′-3′)
	RT-qPCR
AREG Fw	TTGATACTCGGCTCAGGCCAT
AREG Rv	CACAGGGGAAATCTCACTCCC
ASNS Fw	CCTCGCAGGCATGATGAAAC
ASNS Rv	GAAGAAAATCTGGGCGTAAGCA
AURKA Fw	ATATCTCAGTGGCGGACGAG
AURKA Rv	TGAGACCCTCTAGCTGTAATAAGT
B2M Fw	AGGCTATCCAGCGTACTCCA
B2M Rv	ATGGATGAAACCCAGACACA
BIRC5 Fw	AGGACCACCGCATCTCTACA
BIRC5 Rv	TTTCCTTTGCATGGGGTCGT
BAX Fw	CCTGGAGGGTCCTGTACAATCT
BAX Rv	GCACCTAATTGGGCTCCATCT
BMF Fw	CCCTCCTTCCCAATCGAGTCT
BMF Rv	CTCCATCTCTCCTGGGTGACT
CDKN1A Fw	CTGGAGACTCTCAGGGTCGAA
CDKN1A Rv	GATTAGGGCTTCCTCTTGGAG
CHOP Fw	AGAACCAGGAAACGGAAACAGA
CHOP Rv	TCTCCTTCATGCGCTGCTTT
DDIT4 Fw	CTAGCTGCGGCTTCTACGC
DDIT4 Rv	CCAAAGGCTAGGCATGGTGA
GADD45 Fw	TCAGCGCACGATCACTGTC
GADD45 Rv	CCAGCAGGCACAACACCAC
GAPDH Fw	TCCAAAATCAAGTGGGGCGA
GAPDH Rv	AGTAGAGGCAGGGATGATGT
MDM2 Fw	GGCCTGCTTTACATGTGCAA
MDM2 Rv	GCACAATCATTTGAATTGGTTGTC
SESN2 Fw	CTCCTCCTTCGTGTTTGGCT
SESN2 Rv	CTCAAAGCCCCCAGAGTTGT
SCL3A2 Fw	AGCTGGAGTTTGTCTCAGGC
SCL3A2 Rv	GGCCAATCTCATCCCCGTAG
TNFRSF10B Fw	TGACTCATCTCAGAAATGTCAATTCTTA
TNFRSF10B Rv	GGACACAAGAAGAAAACCTTAATGC
TRIB3 Fw	AGACTCGCAGCGGAAGTGG
TRIB3 Rv	CTCGCATCTCGCCCCGTC
sXBP1 Fw	CTGAGTCCGAATCAGGTGCAG
sXBP1 Rv	ATCCATGGGGAGATGTTCTGG
uXBP1 Fw	CAGCACTCAGACTACGTGCA
uXBP1 Rv	ATCCATGGGGAGATGTTCTGG
tXBP1 Fw	TGGCCGGGTCTGCTGAGTCCG
tXBP1 Rv	ATCCATGGGGAGATGTTCTGG
	ChIP
p21 Fw	GTGGCTCTGATTGGCTTTCTG
p21 Rv	CTCCTACCATCCCCTTCCTC

Table S7. Quantification of western blots.

Figure 1		Figure 2	
**Sample**	**p53**	**Sample**	**H2AX**
Vector	NS	DMSO	NS
R175H	1.00	14 μM	NS
Y220C	0.92	28 μM	NS
G245S	1.08	42 μM	NS
R280K	1.24	Etop	1.00
			**p-p53 (Ser15)**
	24h	DMSO	1.00
		SLMP53-2	0.91
	48h	DMSO	1.00
		SLMP53-2	0.78
Figure 3			
**Sample**	**eIF2α**	**Sample**	**p-eIF2α**
DMSO	1.00	DMSO	1.00
28μM	1.14	28μM	3.10
Figure 4			
**HuH-7**		**HuH-7KO**	
**MDM2**			
DMSO	1.00	DMSO	1.00
28μM	3.60	28μM	0.64
**p21**			
DMSO	1.00	DMSO	1.00
28μM	3.08	28μM	1.12
**GADD45**			
DMSO	1.00	DMSO	1.00
28μM	13.24	28μM	0.89
**BAX**			
DMSO	1.00	DMSO	1.00
28μM	2.76	28μM	0.85
**KILLER**			
DMSO	1.00	DMSO	1.00
28μM	1.91	28μM	0.73
**Survivin**			
DMSO	1.00	DMSO	1.00
28μM	0.36	28μM	1.02
**VEGF**			
DMSO	1.00	DMSO	1.00
28μM	0.61	28μM	1.04
Figure 5A			
**Input**		**IP**	
**Sample**	**p53**	**Sample**	**PAb1620**
DMSO	1.00	DMSO	1.00
28μM	0.99	28μM	1.10
42 μM	0.82	42 μM	1.70
		**Sample**	**PAb240**
		DMSO	1.00
		28μM	0.94
		42 μM	0.69
Figure 5B			
**Input**		**IP**	
**Sample**	**Hsp90**	**Sample**	**Hsp90**
DMSO	1.00	DMSO	1.00
28μM	1.11	28μM	0.98
42 μM	0.94	42 μM	1.08

**Sample**	**Hsp70**	**Sample**	**Hsp70**
DMSO	1.00	DMSO	1.00
28μM	1.62	28μM	1.36
42 μM	1.58	42 μM	1.56

**Sample**	**Hsp40**	**Sample**	**Hsp40**
DMSO	1.00	DMSO	1.00
28μM	1.17	28μM	0.81
42 μM	0.89	42 μM	1.09

**Sample**	**p53**
DMSO	1.00
28μM	1.05
42 μM	1.12

NS – no signal.
